# Preclinical evaluation of a regimen combining chidamide and ABT-199 in acute myeloid leukemia

**DOI:** 10.1038/s41419-020-02972-2

**Published:** 2020-09-18

**Authors:** Kai Chen, Qianying Yang, Jie Zha, Manman Deng, Yong Zhou, Guofeng Fu, Silei Bi, Liying Feng, Zijun Y. Xu-Monette, Xiao Lei Chen, Guo Fu, Yun Dai, Ken H. Young, Bing Xu

**Affiliations:** 1 Department of Hematology, Nanfang Hospital, Southern Medical University, 510515 Guangzhou, Guangdong China; 2 Department of Hematology, the First Affiliated Hospital of Xiamen University, 361003 Xiamen, Fujian China; 3 The First People’s Hospital of Foshan (The Affiliated Foshan Hospital of Sun Yat-sen University), 528000 Foshan, Guangdong China; 4 State Key Laboratory of Cellular Stress Biology, Innovation Center for Cell Signaling Network, School of Life Sciences, Xiamen University, 361102 Xiamen, Fujian China; 5 Hematopathology Division and Department of Pathology, Duke University School of Medicine, Duke University Medical Center and Cancer Institute, Durham, NC 27710 USA; 6 Laboratory of Cancer Precision Medicine, Cancer Center, the First Hospital of Jilin University, 130021 Changchun, Jilin China

**Keywords:** Acute myeloid leukaemia, Drug development

## Abstract

Acute myeloid leukemia (AML) is a heterogeneous myeloid neoplasm with poor clinical outcome, despite the great progress in treatment in recent years. The selective Bcl-2 inhibitor venetoclax (ABT-199) in combination therapy has been approved for the treatment of newly diagnosed AML patients who are ineligible for intensive chemotherapy, but resistance can be acquired through the upregulation of alternative antiapoptotic proteins. Here, we reported that a newly emerged histone deacetylase inhibitor, chidamide (CS055), at low-cytotoxicity dose enhanced the anti-AML activity of ABT-199, while sparing normal hematopoietic progenitor cells. Moreover, we also found that chidamide showed a superior resensitization effect than romidepsin in potentiation of ABT-199 lethality. Inhibition of multiple HDACs rather than some single component might be required. The combination therapy was also effective in primary AML blasts and stem/progenitor cells regardless of disease status and genetic aberrance, as well as in a patient-derived xenograft model carrying FLT3-ITD mutation. Mechanistically, CS055 promoted leukemia suppression through DNA double-strand break and altered unbalance of anti- and pro-apoptotic proteins (e.g., Mcl-1 and Bcl-xL downregulation, and Bim upregulation). Taken together, these results show the high therapeutic potential of ABT-199/CS055 combination in AML treatment, representing a potent and alternative salvage therapy for the treatment of relapsed and refractory patients with AML.

## Introduction

Acute myeloid leukemia (AML) is a highly aggressive hematopoietic neoplasm characterized by the clonal expansion of myeloid blasts and impaired hematopoiesis^1^. Refractoriness, relapse, and treatment-related mortality are the major hindrance to AML treatment^2^.

Evasion of apoptosis and enhanced tumor cell survival via dysregulation of Bcl-2 family members is one important therapeutic resistance mechanism^3–5^. ABT-199 (venetoclax), selectively targeting Bcl-2^6^ but not Bcl-xL to avoid thrombocytopenia^7–9^, is highly effective against AML cells in vitro and in vivo, and has shown clinical activity in hematologic malignancies^10–12^. Ω US Food and Drug Administration (FDA) has approved venetoclax plus rituximab for the treatment of patients with relapsed/refractory chronic lymphocytic leukemia carrying 17p deletion^13,14^, and venetoclax in combination with hypomethylating agents (azacitidine and decitabine) or cytarabine for the treatment of newly diagnosed AML patients ineligible for intensive chemotherapy^15,16^. However, resistance to ABT-199 can be acquired from upregulation of alternative antiapoptotic proteins, including the crucial pro-survival protein Mcl-1^17–20^. Mcl-1 overexpression has been associated with high tumor grade and poor survival in cancer^21,22^.

Histone deacetylase inhibitors (HDACi) target histone deacetylases involved in chromatin epigenetic modification, resulting in an open and relaxed chromatin configuration accessible to the transcription machinery^23,24^. CS055 (chidamide) is an oral benzamide-derived HDACi that selectively inhibits HDACs 1, 2, 3, and 10. It has been approved by the Chinese FDA for the treatment of relapsed or refractory peripheral T-cell lymphoma in 2015^25,26^. In previous studies, we have shown the therapeutic potential of CS055 in AML^27,28^. In this study, we sought to test the potential synergistic anti-leukemia effect of a regimen combining CS055 with ABT-199 in AML. It was observed that administration of low-dose CS055 potentiates the cytotoxicity of ABT-199 in vitro in various human AML cell lines and ex vivo in primary AML samples, as well as anti-leukemia efficacy in vivo in a PDX mouse model of AML carrying FLT3-ITD. Mechanistically, CS055 induces DNA double-strand break and alters the balance of pro-apoptotic vs. antiapoptotic Bcl-2 proteins, by which CS055 interacts with ABT-199 to overcome the acquired resistance to ABT-199 in AML without significantly increasing systemic toxicity.

## Materials and methods

### Reagents and cells

Chidamide was supplied by Chipscreen Bioscience Ltd. (Shenzhen, China). ABT-199, Z-VAD-fmk, romidepsin, and vorinostat (SAHA) are all purchased from MedChemExpress (New Jersey, USA).

Molm-13 cells were purchased from AddexBio (San Diego, USA). MV4;11 and NB4 cells were purchased from ATCC (Teddington, UK). OCI-AML2, OCI-AML3 cells were kindly provided by Prof. Bing Z Carter (MD Anderson Cancer Center, USA). All cells were tested and authenticated by an AmpFlSTR Identifiler PCR Amplification Kit (Thermofisher Scientific, USA) in the year of 2018 in our laboratory, and were monthly tested for mycoplasma using PCR method. Peripheral blood samples of healthy donors for hematopoietic stem cell transplantation (*n* = 11) and bone marrow samples of patients with AML (*n* = 36) were obtained from the First Affiliated Hospital of Xiamen University with the informed consent for research purposes only. This study was performed in accordance with the Declaration of Helsinki and approved by the Ethics Review Board of First Affiliated Hospital of Xiamen University.

### Flow-cytometric analysis of apoptosis, cell cycle, and mitochondrial membrane potential (MMP)

For the apoptotic assay, cells were harvested and then subjected to Novocyte (ACEA Bioscience, San Diego, CA, USA) after Annexin V/PI (Thermofisher, USA) staining according to the manufacturer’s instruction. Primary samples with spontaneous apoptosis >40% in the absence of treatment were excluded. To adjust for the variation of spontaneous apoptosis in primary cells, the apoptosis was defined as specific apoptosis^30^. Cell cycle was carried out using the Click-iT EdU Kit (Thermofisher) according to the manufacturer’s instructions. MMP was evaluated by a JC-1 kit (Beyotime Biotechnology, China) as described by the manufacturer.

### Cell viability assay

Cells were plated into 96-well plates and treated with designated drugs for the appropriate time. After treatment, CCK-8 (MedChemExpress, USA) were added. Absorbance at 450 nm was read by a VERSA microplate reader (Molecular Devices, Sunnyvale, CA, USA). IC_50_ values in each cell line were calculated using Compusyn software (ComboSyn Inc., Paramus, NJ, USA).

### Clonogenic assay

For colony-forming assay in vitro, cells were collected, washed, and further cultured in methylcellulose medium (Methocult H4100, Stem Cell Technologies, Vancouver, BC, Canada) at a density of 500 cells/well for 10–14 days, colonies were stained by MTT solution and counted. For tumor-forming capability in vivo, MV4;11 cells were subcutaneously injected into the left flank of nude mice (6-week-old, female, purchased from Xiamen University Animal Center) with the same number of viable cells following 12-hour pre-incubation with drugs. After 2 weeks, subcutaneous tumors were stripped, and maximal diameter (a) and short diameter (b) of the tumor were measured. Tumor volume was calculated using the formula V = (a × b^2^)/2.

### Detection of γH2A.X by confocal microscopy

Cells were collected and fixed with 4% PFA, followed by permeabilization with 0.1% Trition X-100 (Sigma-Aldrich, USA) and block with 5% BSA in PBS, and then incubated with primary antibody against γH2A.X (1:100, CST #9718) overnight at 4 °C. Cells were dropped in glass slides and then mounted using Antifade Mountant with DAPI (Invitrogen, USA) after incubated with secondary antibody (Invitrogen, USA) and phalloidin-FITC (Beyotime Biotechnology, China). Slides were scanned and photographed using a ×100 objective on Zeiss LSM780 confocal microscope (Zeiss, Jena, Germany).

### Alkaline comet assay

Cells were treated and subjected to single-cell gel electrophoresis under alkaline conditions according to the manufacturer’s instructions (Abcam, USA). Using a Nikon TI-U fluorescence microscope, the comets were viewed and analyzed by CASP software (CASP, Wroclaw, Poland). At least 100 comets were counted per condition, and the percentage of DNA in the comet tail was recorded to characterize the DNA damage.

### Immunoblotting

Whole-cell lysates (20 μg of protein/lane) was electrophoresed in 10% SDS-PAGE and transferred to a PVDF membrane (Millipore, Billerica, MA, USA). The transblotted membranes were blocked with 5% nonfat milk in TBS-T and then probed with primary antibodies diluted as recommended by manufacturers, followed by appropriate secondary HRP-conjugated antibody (Merck Millipore, USA). The immunobands were detected with an enhanced ECL substrate (GE Healthcare, Chicago, USA) and visualized using the Amersham Imager 600 (AI600, GE Healthcare, Chicago, USA).

### In vivo study of ABT-199/CS055 efficacy in AML mouse models

All animal studies were performed in accordance with protocols approved by Xiamen University Animal Care and Use Committees. For primary AML-derived xenograft models, freshly isolated bone marrow mononuclear cells (5 × 10^6^ cells) from patient #13 were intravenously transplanted into irradiated (1 Gy) female 6-week-old NOD-Prkdc^−/−^IL2rg^−/−^ mice (NPI, IDMO ltd., Beijing, China) to establish P1 PDX mice after T-cell removal. The hCD45^+^ AML cells in the sick P1 xenograft mice, which account for over 95% of the total, were used for further transplantation. P2 PDX mice were established by intravenously injecting 2 × 10^6^ splenic AML cells from P1 xenograft mice. Three weeks after injection, mice were randomly assigned to four groups (*n* = 10 per group, except *n* = 9 for vehicle group) and treated with vehicle, ABT-199 (100 mg/kg), CS055 (15 mg/kg), or the combination daily for consecutive 4 weeks. Three mice were used for FACS analysis (human CD45, clone HI30, Biolegend; murine CD45 clone 30-F11, Biolegend), and one mouse was used for histological studies from each group after 3-week treatment. The remaining mice were monitored for survival.

### Terminal deoxynucleotidyl transferase-mediated dUTP nick-end labeling (TUNEL) assay

Mouse spleen sections were fixed in neutral buffered containing 10% formalin solution for preparation of tissue slides. TUNEL assays were performed with an In Situ Fluorescein Cell Death Detection Kit (Roche Diagnostics, Mannheim, Germany) according to the manufacturer’s instruction, and visualized using a fluorescent microscope.

### Gene overexpression and knockdown

The open-reading frame of human Mcl-1 cDNA was inserted into the lentiviral transfer vector pLV-EF1a-IRES-EGFP and verified the construction by Sanger sequencing. MCL-1 was knocked down by lentiviral transduction using an MCL-1-specific shRNA transfer vector targeting residues 1117–1135 (shMcl1-1) and 2421-2440 (shMcl1-2) on RefSeq NM_021960.4 in pLV-H1-EF1a-green (Biosettia, San Diego, USA). HDAC1 was knocked down by targeting residues 570–588 (shHDAC1-570) and 1717–1737 (shHDAC1-1717) on RefSeq NM_004964.2. HDAC2 was knocked down by targeting residues 520–538 (shHDAC2-520) and 1761–1781 (shHDAC2-1761) on RefSeq NM_001527.3.

### Statistical analysis

Experiments were performed in triplicate when indicated, and the data were presented as the means ± SD. Statistical analyses were conducted using Prism software v6.0 (GraphPad Software, La Jolla, CA, USA). The log-rank test was used to compare mouse survival curves. For in vitro studies, statistical significance was determined by one-way analysis of variance (ANOVA) followed by LSD posthoc test or two-way ANOVA followed by Bonferroni posthoc test. For in vivo studies, multiple-group comparisons were performed using one-way ANOVA. For the comparison of relationships between various clinical characteristics of AML patients and cytotoxicity of ABT-199/CS055, two-way ANOVA was carried out. Differences with *P* < 0.05 were considered statistically significant.

## Results

### Sublethal doses of CS055 potentiate the activity of ABT-199 in diverse AML cell lines

**Fig. 1:**
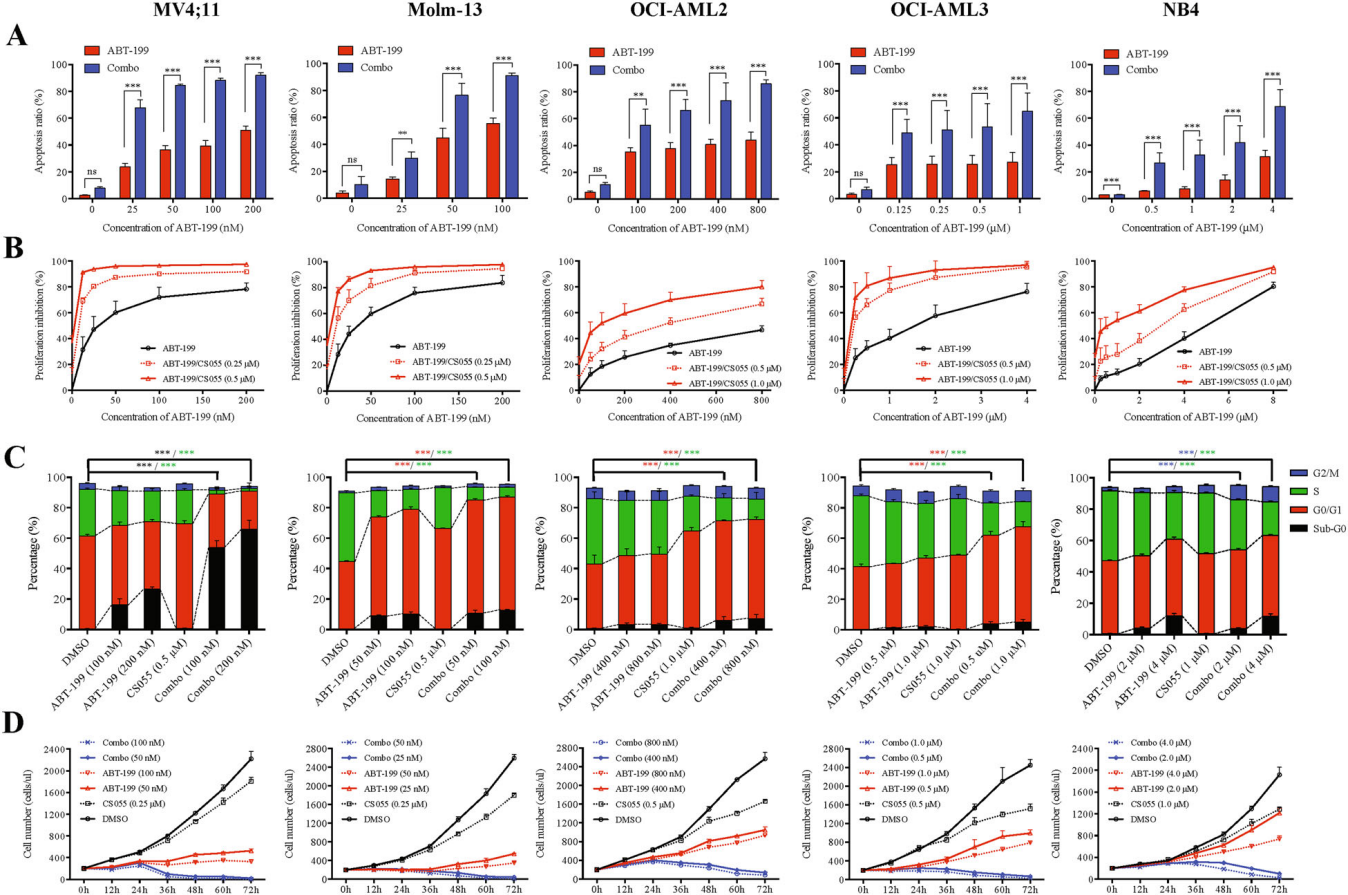
Low and a sublethal dose of CS055 potentiates the anti-leukemic activity of ABT-199 toward various acute myeloid leukemia (AML) cell lines. Five human AML cell lines were exposed to indicated concentrations of ABT-199 ± CS055 (0.5 μM for Molm-13 and MV4;11; 1.0 μM for OCI-AML2, OCI-AML3, and NB4) for 24 or 48 h, after which cells were subjected to the following analyses: **a** the percentage of apoptotic cells were determined at 48 h using Annexin V/PI double staining by flow cytometry. **b** The inhibition rate of cell viability was measured at 48 h using the CCK-8 kit. **c** Cell cycle distribution was assessed at 24 h by flow cytometry (black asterisk, red asterisk, green asterisk, and blue asterisk present the statistical significance of Sub-G0, G0/G1, S, G2/M between DMSO and Combo group, respectively). **d** Cell growth was monitored every 12 h for consecutive 3 days. Values indicate mean ± SD for at least three independent experiments performed in triplicate (*P < 0.05, **P < 0.01, and ***P < 0.001, ns not significant).

We first examined whether the selective HDACi CS055 potentiated the cytotoxicity of ABT-199 in various human AML cell lines after Annexin V/PI staining. Treatment with low doses of CS055 alone did not induce apoptosis compared with the control (*P* > 0.05 for all tested cell lines; Fig. 1a). In contrast, exposure to a series of concentrations of ABT-199 induced marked apoptosis in a dose-dependent manner, while the sensitivity varied among these cell lines (Molm-13 > MV4; 11 > OCI-AML2 > OCI-AML3 > NB4). Notably, co-treatment with CS055 and ABT-199 dramatically increased apoptosis compared with ABT-199 alone in all of the cell lines (*P* < 0.01 for all cases; Fig. 1a). Apoptosis after short exposure (24 h) was also analyzed. As shown in Supplementary Fig. S1A, administration of ABT-199 alone for 24 h significantly induced apoptosis in all cell lines compared with the vehicle controls. While CS055 had no marked activity as a single agent (*P* > 0.05 for all cases), treatment with ABT-199 in combination with CS055 significantly increased apoptosis in MV4;11 and OCI-AML3 cells (*P* < 0.05 for these two lines), but not in Molm-13, OCI-AML2, and NB4 cells.

The effect on cell viability was next assessed. As shown in Fig. 1b, exposure to ABT-199 at various doses as indicated markedly increased inhibition rate of cell viability in a dose-dependent manner. Whereas CS055 alone displayed a modest inhibitory effect, administration of CS055 significantly enhanced ABT-199-mediated inhibition of cell proliferation in all cell lines. Similar results were also obtained with shorter exposure (24 h) in MV4;11 and OCI-AML3 cells, and to a lesser extent in Molm-13, OCI-AML2 and NB4 at relatively higher doses of CS055 (Supplementary Fig. S1B). IC_50_ values were calculated for ABT-199 when administrated alone and in combination with CS055. As shown in Supplementary Table S1, the IC_50_ values of ABT-199 were reduced at least twofolds when co-treatment with CS055 for 48 h, compared with ABT-199 alone in all of these cell lines.

### The combination of ABT-199 and CS055 alters cell cycle distribution and inhibits cell growth in vitro

To further characterize the role of CS055 in enhancing ABT-199-mediated cytotoxicity, we then evaluated the cell cycle status. In all tested AML cell lines, the percentage of cells in the S phase was significantly decreased after short exposure (24 h) to CS055 or ABT-199 alone, while this effect was dramatically aggravated after combined treatment (Fig. 1c). However, the pattern for cell cycle arrest varied among these cell lines. For instance, co-treatment resulted in marked cell cycle arrest at the G0/G1 phase in Molm-13, OCI-AML2, and OCI-AML3 cells, while induced G2/M arrest in NB4 cells. In MV4;11 cells, 24-h exposure to ABT-199/CS055 markedly increased the sub-G0 fraction of cells, consistent with early induction of apoptosis in this line. In line with the remarkable ability of the combined treatment to induce cell cycle arrest, cell growth curves were then examined by assessing the absolute cell numbers every 12 h. Whereas treatment with ABT-199 but not CS055 alone sharply inhibited cell growth compared with the control (Fig. 1d), combined treatment almost completely suppressed the growth of AML cells in all tested cell lines (particularly in Molm-13 and MV4;11 cells).

### CS055 shows a superior resensitization effect than romidepsin in potentiation of ABT-199 lethality in AML cells

**Fig. 2:**
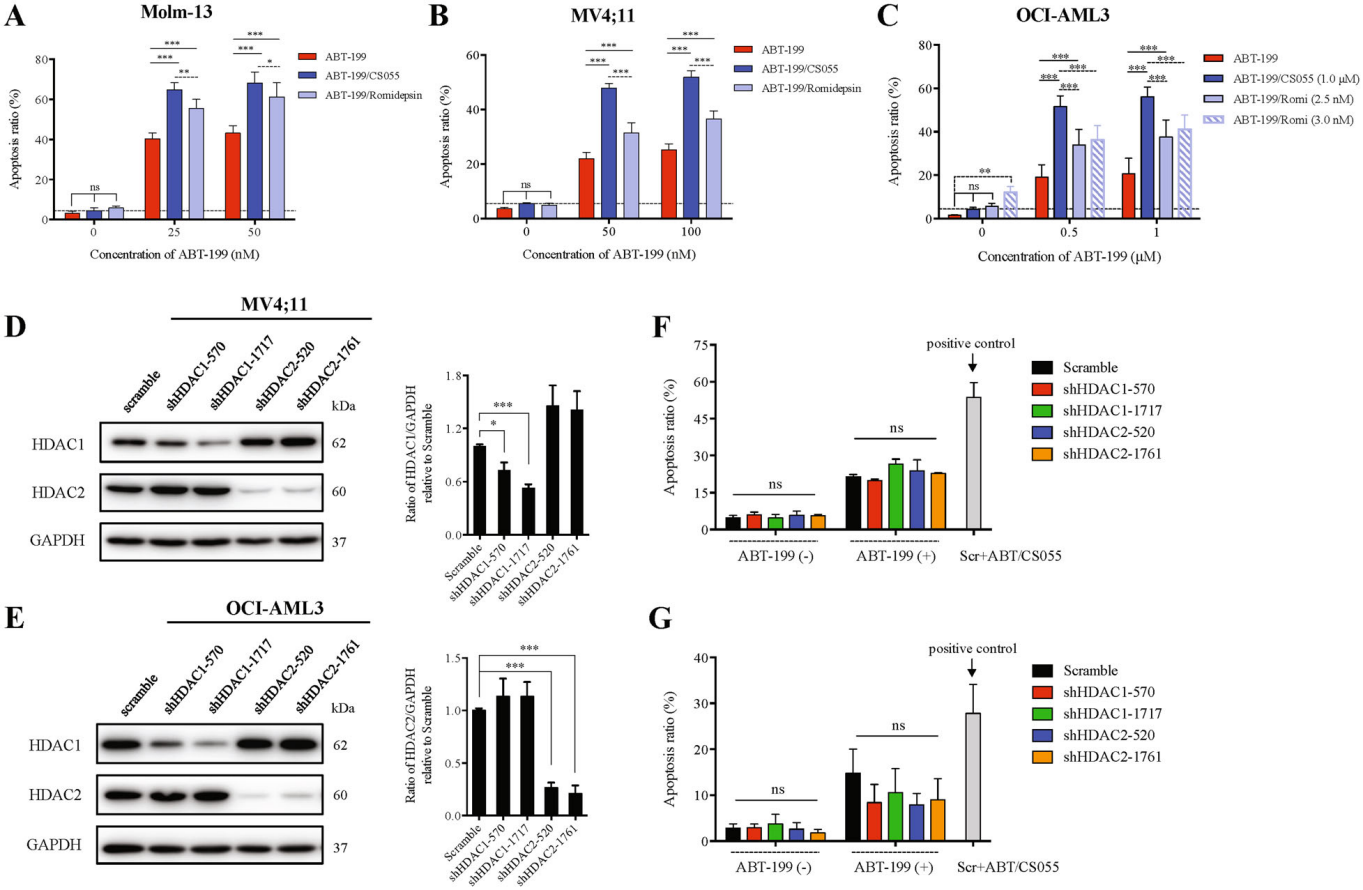
CS055 shows a superior resensitization effect than romidepsin in potentiation of ABT-199 lethality in acute myeloid leukemia (AML) cells. **a** Molm-13, **b** MV4;11, and **c** OCI-AML3 cells were exposed to the indicated concentrations of ABT-199 ± CS055 (0.25 µM for Molm-13 and MV4;11, and 1.0 µM for OCI-AML3) or romidepsin (1.5 nM for Molm-13 and MV4;11, and 2.5–3 nM for OCI-AML3) for 48 h, after which the percentage of apoptotic cells was determined using Annexin V/PI double staining by flow cytometry. **d**, **e** Western blot analysis was performed to validate downregulation efficiency of HDAC1 and HDAC2 in MV4;11 and OCI-AML3 cells. **f**, **g** Cells were exposed to ABT-199 (50 nM for MV4;11, 1.0 µM for OCI-AML3), and then the percentage of apoptotic cells was determined by flow cytometry. Scramble shRNA-transfected cells exposed for 48 h to combined treatment with ABT-199 (50 nM for MV4;11, 1.0 µM for OCI-AML3) and CS055 (0.5 µM for MV4;11, 1.0 µM for OCI-AML3) serve as a positive control. Values indicate mean ± SD for at least three independent experiments performed in triplicate (*P < 0.05, **P < 0.01, ***P < 0.001, ns not significant).

We then compared CS055 with romidepsin (also known as istodax, depsipeptide, or FK228), a selective inhibitor of HDAC1 and HDAC2 that has been approved for the treatment of cutaneous T-cell lymphoma^29^, in potentiation of ABT-199 activity. Sublethal doses of CS055 and romidepsin were used to yield comparable single-agent activity (*P* > 0.05 for CS055 vs romidepsin alone in all three lines; Fig. 2a–c). Whereas romidepsin also interacted with ABT-199 to induce apoptosis, CS055 exhibited the greater capacity to potentiate lethality of ABT-199 in Molm-13 and MV4;11 (Fig. 2a, b), as well as in OCI-AML3 (Fig. 2c), an AML cell line that is more resistant to ABT-199^30^. To understand whether inhibition of pan HDACs or specific HDAC(s) would be better to sensitize AML cells to ABT-199, HDAC1 and HDAC2 were knocked down in MV4;11 and OCI-AML3 cells (Fig. 2d, e). However, knockdown of either HDAC1 or HDAC2 failed to increase the sensitivity of ABT-199 in MV4;11 (Fig. 2f) and OCI-AML3 cells (Fig. 2g). In addition, we also compared the action of CS055 to another pan-HDAC inhibitor SAHA (also known as vorinostat, approved by FDA for treatment of cutaneous T-cell lymphoma). Consistent with our previous observations on CS055, sublethal doses of vorinostat also markedly potentiates apoptosis induced by ABT-199 (*P* < 0.001 for combo vs ABT-199 alone), while the action of SAHA seems greater than CS055 in OCI-AML3 cells (new Supplementary Fig. S2). Taken together, these results indicate that low (sublethal) doses of CS055 remarkably potentiates the anti-leukemia activity of ABT-199. They also raise a possibility that pan-HDAC inhibitors (e.g., CS055 and SAHA) might be superior to HDAC isoform-selective inhibitors (e.g., romidepsin) in potentiation of ABT-199 lethality toward AML cells.

### Co-administration of ABT-199 and CS055 impairs colony- and tumor-forming capabilities in vitro and in vivo

**Fig. 3:**
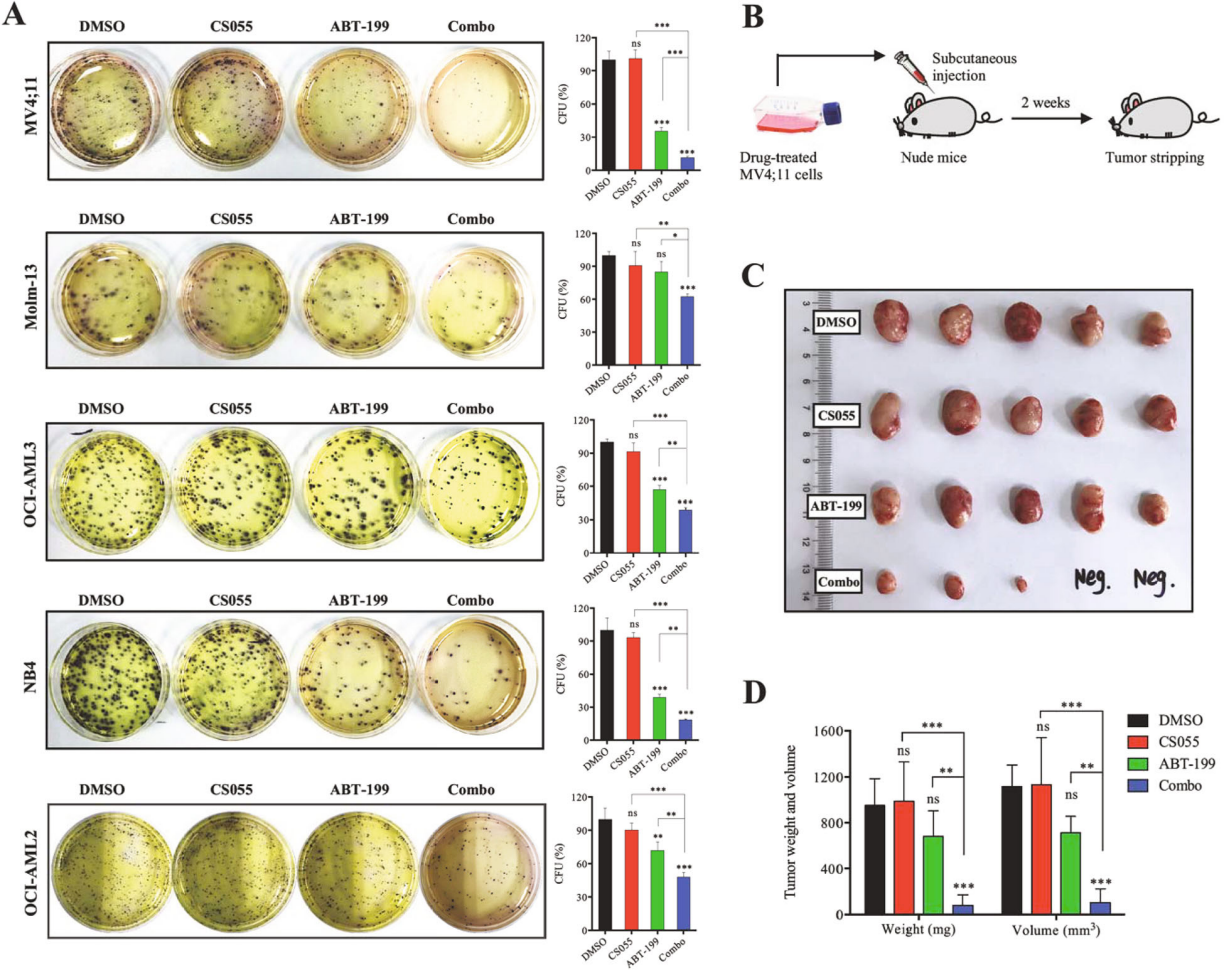
ABT-199 in combination with CS055 impairs colony- and tumor-forming ability of acute myeloid leukemia (AML) cells in vitro and in vivo. **a** AML cells were treated with ABT-199 (200 nM for MV4;11, 100 nM for Molm-13, 800 nM for OCI-AML2, 1 μM for OCI-AML3, and 4 μM for NB4) ± CS055 (0.5 μM for MV4;11/Molm-13, and 1.0 μM for the other lines) for 12 h, after which the clonogenicity assay was performed to determine the percentage of CFU (left, representative images; right, bar graphs). Values indicate mean ± SD for at least three independent experiments performed in triplicate (*P < 0.05, **P < 0.01, and ***P < 0.001; ns not significant for CS055 vs vehicle). **b** The scheme for the procedure of the in vivo experiment. MV4;11 cells were pre-incubated for 12 h with ABT-199 ± CS055, followed by subcutaneous injection on the left flank of nude mice. **c**, **d** Two weeks after cell inoculation, tumors were removed (**c**) and measured for weight and size (**d**).

We next examined whether the combination of CS055 and ABT-199 affects the clonogenicity of AML cells. As shown in Fig. 3a, the colony-forming assay revealed that 12-hour pretreatment with ABT-199 alone moderately but significantly inhibited colony formation in all tested cell lines except Molm-13 cells (*P* < 0.01), while a low dose of CS055 had no clear effect (*P* > 0.05 for all cell lines). Notably, this effect of ABT-199 was sharply enhanced when combined with CS055 (*P* < 0.001). We then further examined the tumor-forming capability of drug-treated AML cells in a nude mouse xenograft model, as depicted in Fig. 3b. Interestingly, unlike the marked inhibitory effect of ABT-199 on colony-forming ability in vitro, pretreatment with ABT-199 did not significantly affect tumor-forming capacity of MV4;11 cells (Fig. 3c, d). Nevertheless, pretreatment with the combination resulted in a significant reduction in tumor size and weight (Fig. 3c, d). In addition, it was noted that AML cells pre-treated with combination treatment failed to form tumors in two of five mice (Supplementary Fig. S3).

### CS055 potentiates apoptosis induced by ABT-199 via dysregulation of anti- and pro-apoptotic Bcl-2 family proteins

**Fig. 4:**
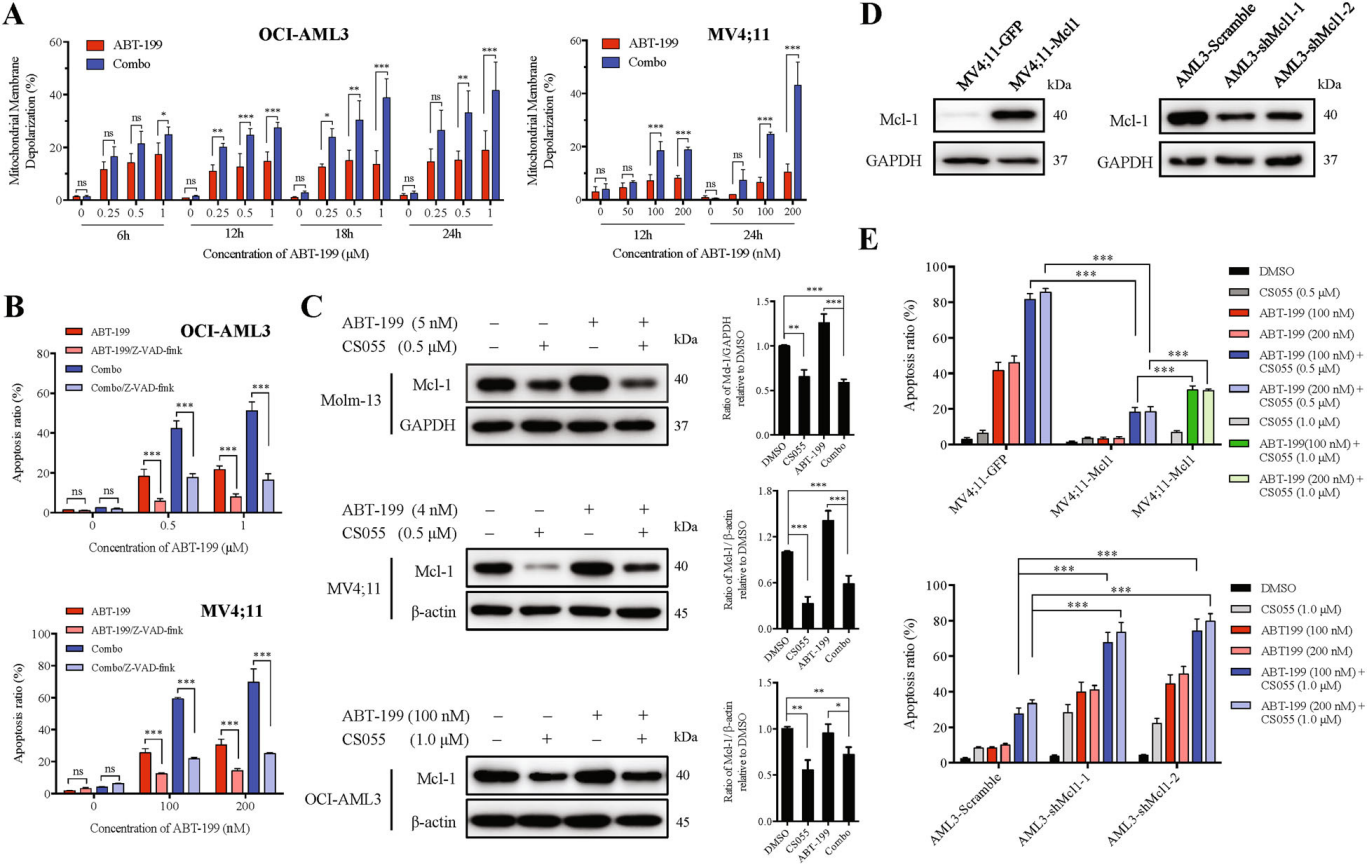
CS055 enhances mitochondrion-dependent apoptosis induced by ABT-199 by downregulation of Mcl-1. **a** OCI-AML3 (left) and MV4;11 cells (right) were exposed to the indicated concentrations of ABT-199 ± CS055 (1.0 μM for OCI-AML3; 0.5 μM for MV4;11) for 6–24 h, after which mitochondrial membrane potential (MMP) was measured using the JC-1 kit by flow cytometry. **b** OCI-AML3 (upper) and MV4;11 cells (lower) were pre-incubated with 20 μM Z-VAD-fmk for 2 h, followed by treatment with indicated concentrations of ABT-199 ± CS055 (1.0 μM for OCI-AML3; 0.5 μM for MV4;11) for additional 24 h. The percentage of apoptosis was then assessed by flow cytometry. **c** Western blot analysis was performed to monitor the expression of Mcl-1 in Molm-13, MV4;11, and OCI-AML3 cells treated with the indicated concentration of ABT-199 ± CS055. Blots were probed for β-actin or GAPDH as loading controls. **d** Mcl-1 was ectopically expressed in MV4;11 cells (left) or knocked down by shRNA in OCI-AML3 cells (right). **e** MV4;11 cells overexpressing Mcl-1 (upper) and OCI-AML3 cells with Mcl-1 knockdown (lower) were treated with indicated concentrations of ABT-199 ± CS055 for 48 h, after which the percentage of apoptosis was determined by flow cytometry. For panels **a**, **b** and **e**, values indicate mean ± SD for at least three independent experiments performed in triplicate (*P < 0.05, **P < 0.01, and ***P < 0.001, ns not significant).

To explore the mechanism of action for the ABT-199/CS055 combination regimen, MMP was analyzed. As shown in Fig. 4a (OCI-AML3, left; MV4;11, right), combined treatment in AML cells resulted in a marked increase in the fraction of cells with mitochondrial membrane depolarization compared to every single agent. The pan-caspase inhibitor Z-VAD-fmk was then employed to determine the role of caspase activation, an event downstream of mitochondrial injury in the intrinsic apoptotic cascade. Indeed, the addition of Z-VAD-fmk (20 μM) significantly prevented apoptosis induced by either ABT-199 alone or in combination with CS055 in AML cells (*P* < 0.01 for treatment with vs without Z-VAD-fmk, Fig. 4b).

As Mcl-1 plays a crucial role in ABT-199 resistance, we thus examine the function of Mcl-1 in the lethality of the ABT-199/CS055 combination. Western blot analysis revealed that while treatment with ABT-199 resulted in a modest increase in the protein level of Mcl-1, exposure to CS055 in the presence or absence of ABT-199 led to a significant reduction in Mcl-1 expression in OCI-AML3, Molm-13, and particularly MV4;11 cells (Fig. 4c; *P* < 0.05 vs. untreated control or ABT-199 alone, respectively). Next, the functional contribution of Mcl-1 to ABT-199/CS055 lethality was then examined. Ectopic expression of Mcl-1 in MV4;11 cells (Fig. 4d) conferred resistance to both ABT-199 alone (red columns) and combined ABT-199/CS055 treatment (blue columns), which could be partially reversed by increasing dose of CS055 (e.g., from 0.5 μM to 1.0 μM; green columns) as shown in Fig. 4e (upper). The results also raise the possibility that increasing dose of CS055 might partially overcome ABT-199 resistance in AML cells. In contrast, shRNA knockdown of Mcl-1 (Fig. 4d) sensitized OCI-AML3 cells to ABT-199 and CS055 as a single agent as well as their combination (Fig. 4e, lower).

In addition, the pro-apoptotic and other anti-apoptotic proteins were also examined. Notably, exposure to CS055 in the presence or absence of ABT-199 also resulted in a significant increase in the protein level of Bim, but a modest decrease of Bcl-xL in OCI-AML3 and MV4;11 cells (Supplementary Fig. S4). Together these findings suggest that the activity of CS055 to potentiate ABT-199 lethality might stem from the altered balance between pro-apoptotic (e.g., Bim) and anti-apoptotic proteins (e.g., Mcl-1 and Bcl-xL) of the Bcl-2 family.

### CS055 induces DNA damage in AML cells, an event likely not enhanced by ABT-199

**Fig. 5:**
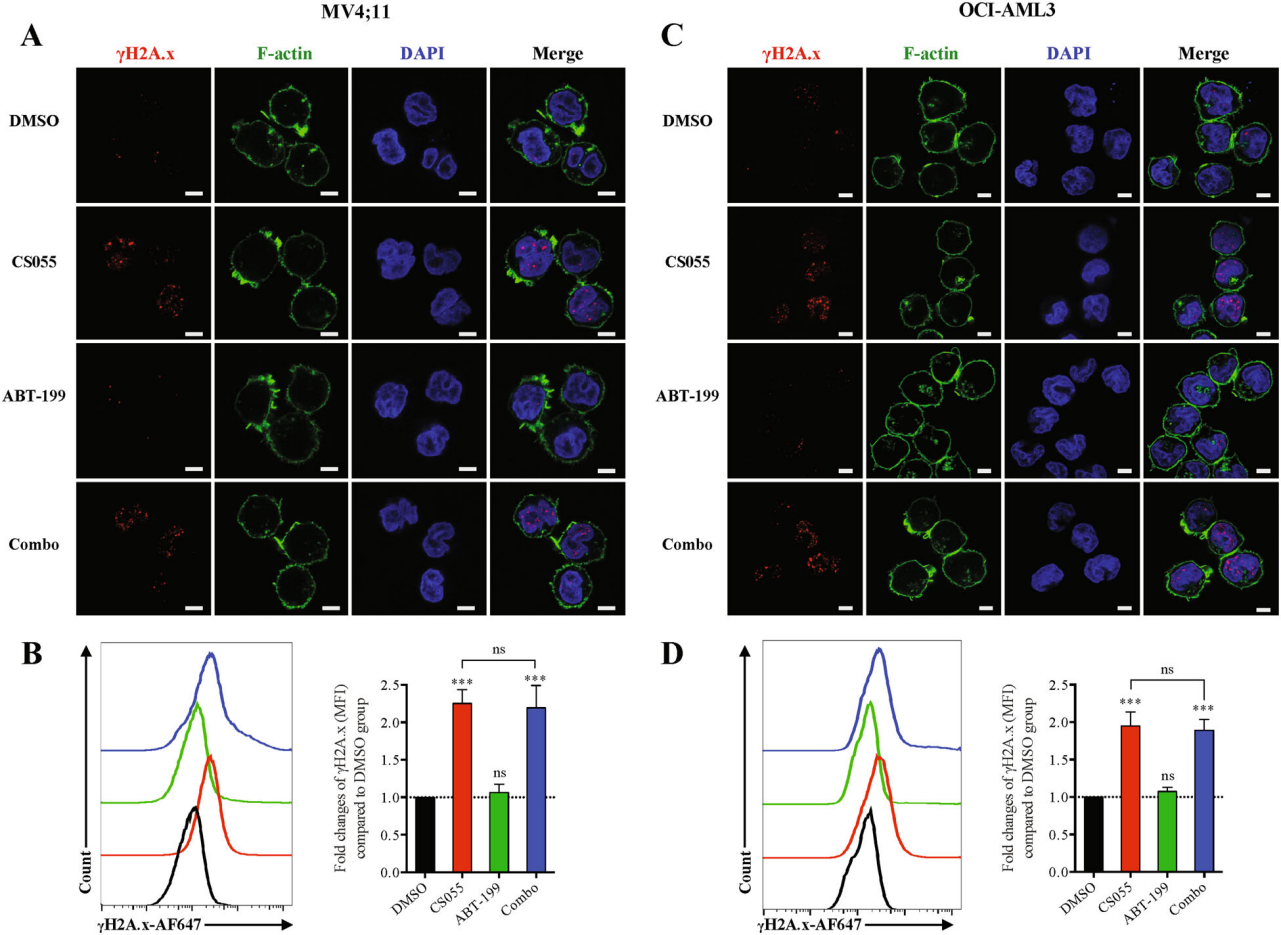
CS055 induces expression and foci formation of γH2AX, an event not enhanced by ABT-199. MV4;11 (**a**, **b**) and OCI-AML3 cells (**c**, **d**) were treated with ABT-199 (5 nM for MV4;11, 100 nM for OCI-AML3) ± CS055 (0.5 μM for MV4;11, 1.0 μM for OCI-AML3) for 18 h, after which cells were subjected to immunofluorescent staining for Ser139 phosphorylation of histone H2A.X (H2A.X, red) and confocal microscopy (**a**, **c**; phalloidin—green, DAPI—blue; scale bars: 5 μm.) or flow cytometry for monitoring H2A.X expression (**b**, **d**). Values indicate mean ± SD for at least three independent experiments performed in triplicate (***P < 0.001, ns not significant).

DNA damage is considered as one of the most important mechanisms underlying the cytotoxicity of numerous anticancer agents^31,32^, including HDAC inhibitors^33^. We thus examined whether DNA damage would contribute to enhanced cytotoxic effects of the combination. To this end, γH2AX (a serine 139 phosphorylated form of histone H2AX), a marker of DNA double-strand breaks^34^, was detected. As shown in Fig. 5, exposure to a sublethal dose of CS055 alone resulted in a marked increase in the number of γH2AX foci and in the percentage of cells with increased γH2AX immunofluorescence in MV4;11 (Fig. 5a, b) and OCI-AML3 cells (Fig. 5c, d). However, administration of ABT-199 did not further increase γH2AX foci number and expression in AML cells exposed to CS055 (Fig. 5a–d). These results were further confirmed by the comet assay (Supplementary Fig. S5). Moreover, although ectopic expression of Mcl-1 prevented apoptosis induced by the CS055/ABT-199 combination, it failed to prevent DNA damage triggered by CS055 in MV4;11 cells (Supplementary Fig. S6A). Similarly, shRNA knockdown of Mcl-1 also did not affect γH2AX expression induced by CS055 + /− ABT-199 in OCI-AML3 cells (Supplementary Fig. S6B).

### The combination regimen of CS055 with ABT-199 is effective against primary AML blasts and stem/progenitor cells while sparing normal hematopoietic progenitors

**Fig. 6:**
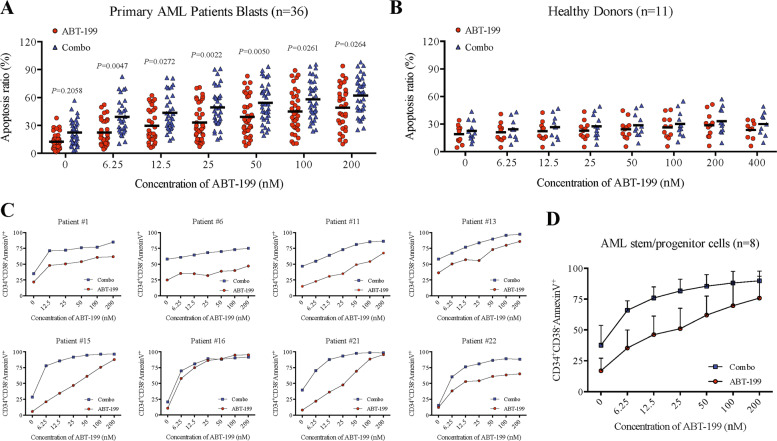
ABT-199/CS055 combination regimen is effective toward primary acute myeloid leukemia (AML) blasts and leukemic stem/progenitor cells while sparing normal hematopoietic progenitors. **a**, **b** Mononuclear cells isolated from bone marrow samples of patients with AML (**a**; *n* = 36) and peripheral blood of healthy donors (**b**; *n* = 11) were exposed to the indicated concentrations of ABT-199 ± 1.0 μM CS055 for 48 h, after which flow-cytometric analysis was performed to determine the percentage of Annexin V^+^ apoptotic cells. Each dot represents one individual. **c**, **d** After treatment with the indicated concentrations of ABT-199 ± 1.0 μM CS055 for 48 h, the percentage of apoptotic cells in primary CD34^+^CD38^−^ AML stem/progenitor cells was determined by flow cytometry in eight patients (#1, #6, #11, #13, #15, #16, #21, and #22 as shown in Supplementary Table S2) who had enough number of CD34^+^CD38^−^ cells available for this analysis (**c**). The results obtained from these eight patients were then analyzed together (**d**).

To further validate the activity of the ABT-199/CS055 combination against AML cells, primary leukemic blasts were employed. Clinical characteristics of patients are summarized in Supplementary Table S2. As shown in Fig. 6a, ex vivo treatment with ABT-199 alone resulted in a marked increase in apoptosis of primary AML blasts, although the responses varied among patient samples. However, exposure to a low dose of CS055 alone (e.g., 1 μM) was unable to induce apoptosis (*P* = 0.2058 for CS055 vs vehicle). Of note, combined treatment with ABT-199 and CS055 led to a greater increase in apoptosis than ABT-199 alone. We then evaluated the relationship between clinical characteristics of patients and the sensitivity of the ABT-199/CS055 combination. As shown in Supplementary Table S3, the sensitivity to this combination regimen did not significantly differ (*P* > 0.05) between different FAB subtypes, risk status, and genetic aberrations, except for disease types (secondary AML were less sensitive than primary AML, *P* = 0.002) and hyperleukocytosis (WBC > 100 × 10^9^/L were more sensitive, *P* = 0.0323). In sharp contrast, identical treatments had almost no effect on normal hematopoietic cells isolated from healthy donors (*P* > 0.05, Fig. 6b). Moreover, AML stem/progenitor cells, which characterized by the CD34^+^CD38^−^ immunophenotype and known to be highly resistant to conventional chemotherapy^35^, were analyzed in primary samples of eight AML patients. The percentage of apoptotic cells in the population of CD34^+^CD38^−^ cells was determined using a gating strategy shown in Supplementary Fig. S7. As shown in Fig. 6c, d, combined treatment with ABT-199 and CS055 led to a significant increase in apoptosis of CD34^+^CD38^−^ cells compared to ABT-199 alone in seven of eight patient samples, except patient #16 whose CD34^+^CD38^−^ cells were extremely sensitive to ABT-199 alone.

### The ABT-199/CS055 regimen is active in vivo in a patient-derived xenograft (PDX) mouse model of AML carrying FLT3-ITD

**Fig. 7:**
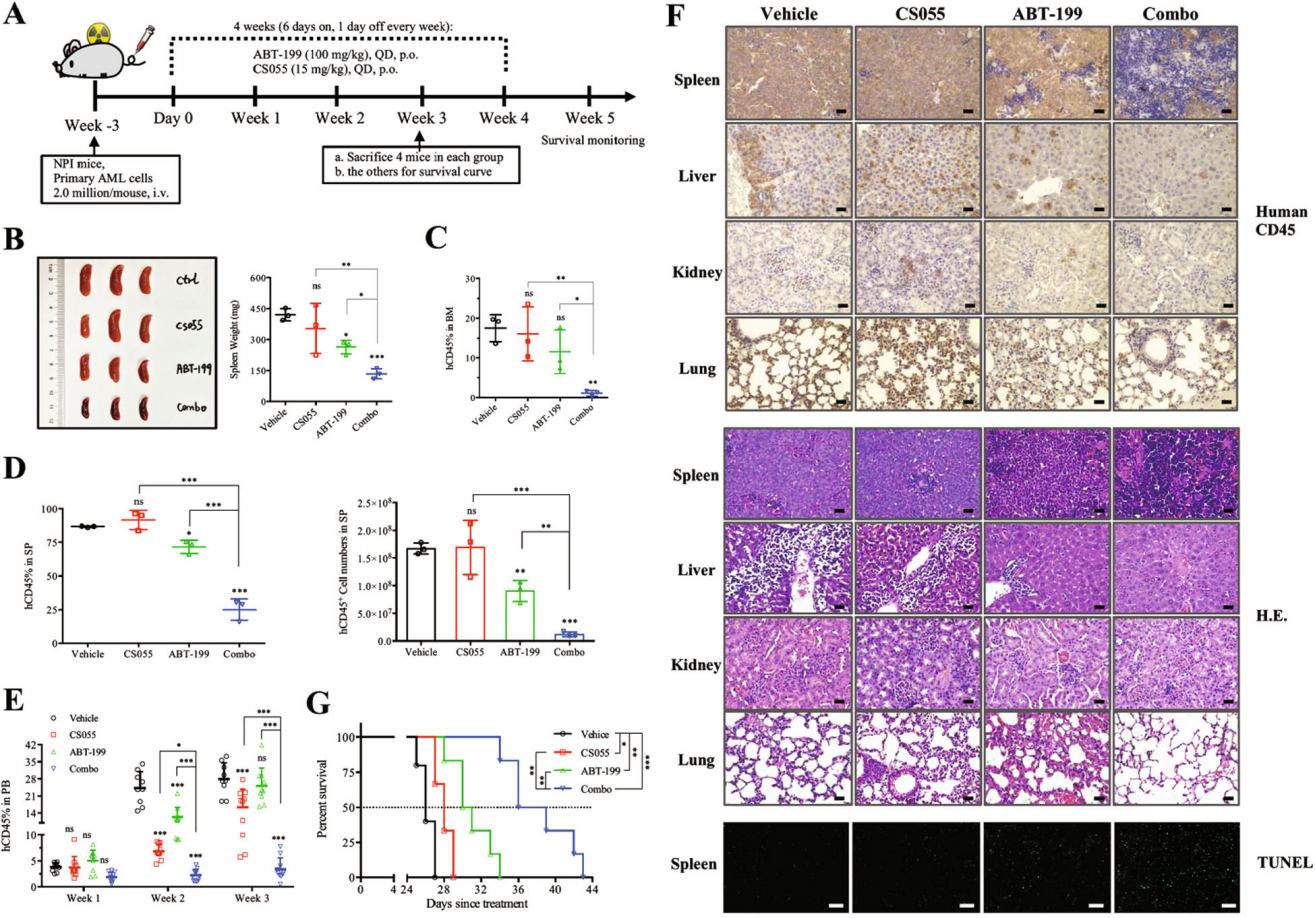
The ABT-199/CS055 combination regimen is highly active in vivo in a PDX mouse model generated from an acute myeloid leukemia (AML) patient carrying FLT3-ITD mutation. **a** The scheme for the process of the experiments using a PDX mouse model generated by tail vein injection of NOD-Prkdc^−/−^IL2rg^−/−^ mice with cells obtained from a patient carrying FLT3-ITD (#13 as shown in Supplementary Table S2). Three weeks after cell inoculation, mice were randomly assigned into four groups and received vehicle, CS055 (15 mg/kg), ABT-199 (100 mg/kg), or the combination by oral gavage for four consecutive weeks by following a weekly schedule of 6 days on and 1 day off. **b** Images of spleens removed from three representative mice were shown (left), and the weight of the spleens was measured (right). **c**–**e** Flow-cytometric analysis was performed to determine tumor burden of human CD45^+^ leukemic cells in femur bone marrow (BM; **c**), and spleen (SP; **d**, left— percentage and right—absolute number of human CD45^+^ leukemic cells). **e** Flow-cytometric analysis was performed to monitor the percentage of human CD45^+^ leukemic cells in peripheral blood (PB) every week. **f** Immunohistochemical staining for human CD45 (upper) and H&E staining (middle) were performed to examine infiltration of tumor cells in the spleen, liver, kidney, and lung (scale bar: 25 µm). Alternatively, TUNEL staining was performed to visualize apoptotic cells in the spleen (bottom; scale bar: 100 µm. **g** Kaplan–Meier analysis was performed to assess animal survival (**P < 0.01).

AML patients carrying FLT3-ITD have a poor prognosis as indicated in the NCCN guidelines Version 1.2016 Acute Myeloid Leukemia. To address whether this combinational regimen would be active against FLT3-ITD-positive AML, we generated a PDX model by inoculating cells obtained from patient #13 who carried FLT3-ITD. Mice were randomly assigned into four groups and subjected to treatment following a weekly schedule of 6 days on and 1 day off for four consecutive weeks (Fig. 7a). After 3 weeks of treatment, four mice from each treatment group were sacrificed for measurement of tumor burden, while the remaining mice were used for survival analysis. As shown in Fig. 7b, treatment with ABT-199 but not CS055 alone moderately reduced spleen size (*P* = 0.0193 for ABT-199 vs vehicle; *P* > 0.05 for CS055 vs vehicle). Of note, combined treatment dramatically ameliorated the disease-associated splenomegaly (*P* = 0.0007 for combination vs vehicle). FACS analysis revealed that combination treatment remarkably reduced tumor burden, reflected by significantly less human CD45^+^ cells in murine bone marrow (BM, Fig. 7c), spleen (SP, Fig. 7d), and peripheral blood (PB, Fig. 7e). Immunohistochemical analysis in the spleen, liver, kidney, and lung confirmed that the combined treatment significantly decrease the tumor burden (Fig. 7f and Supplementary Fig. S8). TUNEL staining of spleen sections revealed a significant increase in apoptosis after co-treatment (Fig. 7f, bottom). Last, whereas treatment with ABT-199 as single agent modestly improved survival of mice, co-administration of ABT-199/CS055 substantially prolonged animal survival (Fig. 7g).

## Discussion

Although conventional therapies combining multiple chemotherapeutic drugs and hematopoietic stem cell transplantation have greatly improved the survival of many patients with AML, this disease still remains incurable. Thus, new agents and drug combinations with less systemic toxicity are urgently needed. Bcl-2 inhibitors directly targeting the regulatory machinery of apoptosis^30^, as well as epigenetic therapy^36^ (including HDAC inhibitors), represent two promising alternative strategies for the treatment of myeloproliferative neoplasms, which may overcome resistance toward conventional chemotherapeutic drugs. In this preclinical study, we found a regimen combining Bcl-2 inhibitor ABT-199 with low dose of the HDACi (CS055), both of which have been approved for the treatment of certain hematologic malignancies, displayed the superb anti-leukemic activity against AML cells and CD34^+^/CD38^−^ leukemic stem/progenitor cells, whereas largely sparing normal hematopoietic progenitors. Moreover, this combination regimen was effective across a spectrum of AML subtypes carrying diverse genetic alterations in the in vitro, ex vivo, and in vivo settings. Notably, the observation that the ABT-199/CS055 combination was highly active in a PDX mouse model of AML carrying FLT3-ITD highlights the promising activity of this regimen for the treatment of high-risk AML patients with poor prognosis. Therefore, the regimen combining ABT-199/CS055 may represent an effective therapy to treat AML.

**Fig. 8:**
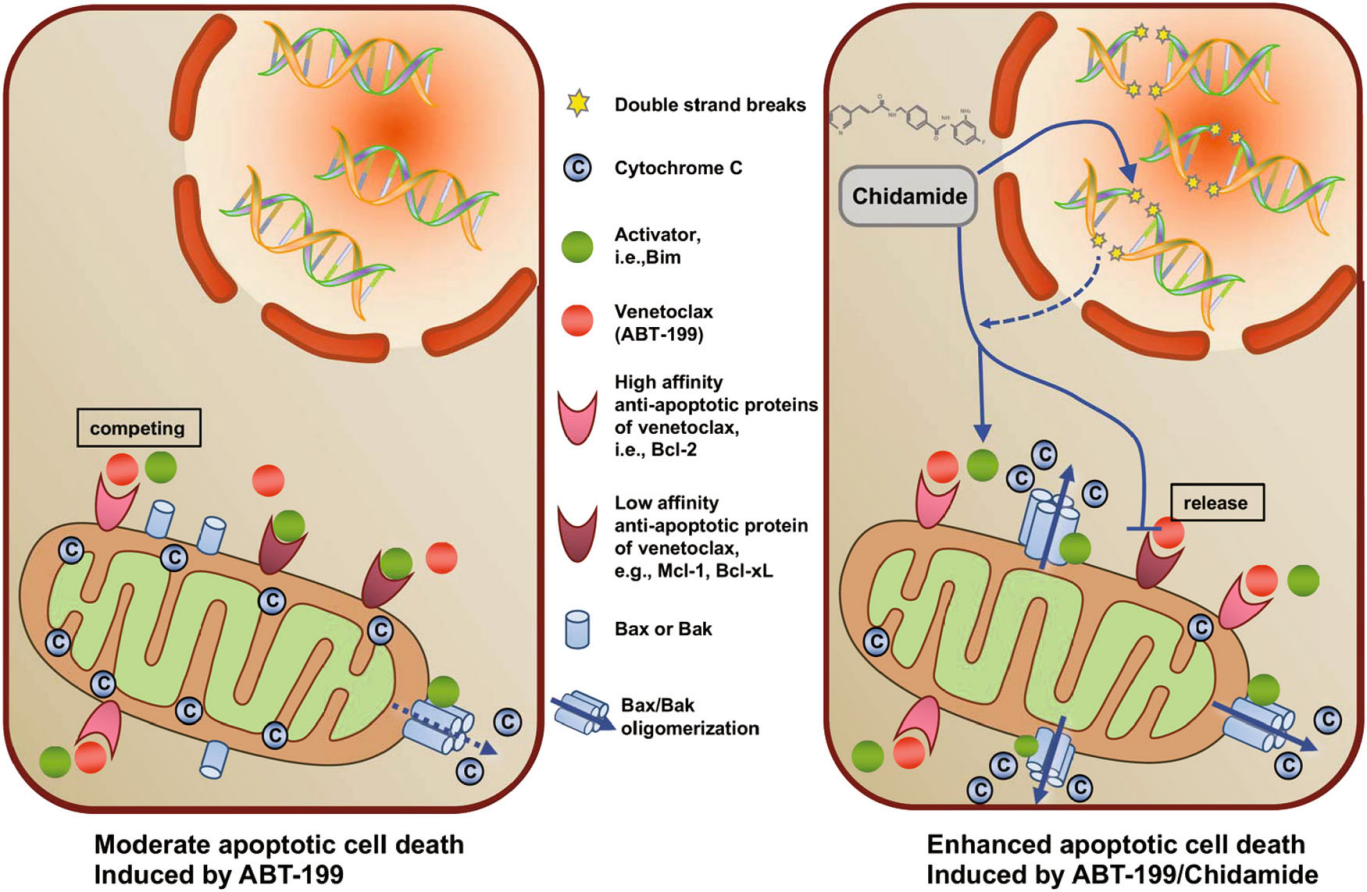
Mechanism of action of the combined treatment. CS055 induces DNA double-strand break accumulation and alters the balance of pro-apoptotic vs. antiapoptotic Bcl-2 proteins, by which CS055 interacts with ABT-199 to overcome the acquired resistance to ABT-199 in acute myeloid leukemia (AML).

Adaptive drug resistance is common for targeted therapies, although the patients respond well to initial treatment^37^. Among various mechanisms underlying adaptive resistance to ABT-199, Mcl-1 represents a key compensatory factor that acts to re-sequester the pro-apoptotic Bim released from Bcl-2 by ABT-199^18–20^. In this study, we found that exposure to low (sublethal) doses of CS055 resulted in marked Mcl-1 and Bcl-xL downregulation, and Bim upregulation. Moreover, ectopic expression of Mcl-1 functionally protected AML cells from apoptosis induced by the combination, while knockdown of Mcl-1 significantly sensitized AML cells to ABT-199 and CS055 administrated individually or in combination. Thus, these findings support the notion that Mcl-1 may play a role in resistance to ABT-199. They also raise a potential mechanism for the interaction between CS055 and ABT-199, in which while CS055 induces Bim expression, inhibition of Bcl-2 by ABT-199 and downregulation of Mcl-1 and Bcl-xL by CS055 could fully unleash and activate Bim to induce apoptosis and probably to overcome ABT-199 resistance in AML cells (Fig. 8).

DNA damage response is essential for the maintenance of genomic integrity while often dysregulated in tumor cells, resulting in genomic instability, a hallmarks of cancer^32^. Consequently, chemotherapeutic agents (e.g., danuorubicin and cytarabine, the mainstay of AML treatment) cause excessive DNA damages beyond the DNA repair capacity of the cell and thus activate downstream programmed cell death, including intrinsic apoptotic death. In this study, we found that treatment with CS055, but not ABT-199, resulted in marked DNA double-strand breaks in AML cells. In contrast, ABT-199 neither induced γH2AX foci formation nor enhanced DNA damage induced by CS055. Moreover, while ectopic expression of Mcl-1 markedly blocked apoptosis induced by ABT-199 alone or the combination, it failed to prevent DNA damage triggered by CS055 or in combination with ABT-199. These observations argue against a notion that DNA damage may play a role in potentiation of ABT-199 lethality by CS055 regardless of the status of Mcl-1 (Fig. 8). Given that DNA damage signals might in turn evoke the downstream apoptotic cascade if DNA repair fails, therefore, the possibility that CS055-mediated DNA damage might lower down the threshold (termed priming) for ABT-199 and potentially induce the activation of the downstream intrinsic apoptotic pathway cannot be excluded.

The bulk of preclinical evidence supports the promising activity of the ABT-199/CS055 combination regimen for the treatment of AML, particularly those hard-to-treat subtypes. AML carrying FLT3-ITD mutation has been recognized as a high-risk feature that is unfavorable for conventional chemotherapy^38^, and patients with this subtype of AML should be considered for clinical trials where available. In this context, we observed that ABT-199/CS055 combination regimen was highly active in human AML cell lines carrying FLT3-ITD (e.g., MV4;11 and Molm-13), as well as primary leukemic blasts from patients with FLT3-ITD. Of note, the efficacy of this regimen was further replicated in a PDX model, an approach substantially used for preclinical evaluation of personalized treatment strategies^39,40^, which was generated from an FLT3-ITD patient who was relapsed after two cycles of MD-Arac chemotherapy. Moreover, this regimen displayed with a well-tolerated safety profile, manifested by no marked hair loss or decreased mobility of mice (data not shown). Taken together, these findings strongly suggest that the ABT-199/CS055 combination regimen might represent an effective therapy for the treatment of AML patients carrying high-risk FLT3-ITD, probably other poor-prognostic genetic alterations as well, although future studies are warranted to include more PDX models.

Interestingly, the ex vivo efficacy of combined treatment is significantly associated with peripheral WBC count at diagnosis, implying that the combination regimen could rapidly and potently diminish peripheral tumor burden in AML patients. In addition, we also found that patients with secondary AML (MDS-AML or CMML-M5) were less responsive to the combined treatment, which might attribute to the fact that therapeutic strategies for myeloproliferative neoplasms were more dependent on epigenetic therapy^41^. It might be beneficial if we employed a higher dose of epigenetic drug HDACi rather than sublethal dose, although it needs to be further examined.

In summary, this study provides strong preclinical evidence supporting that the regimen combining CS055 and ABT-199 is highly effective towards high-risk AML with diverse cytogenetic and genetic aberrations, including FLT3-ITD, as well as refractory/relapse diseases. Therefore, the novel combination regimen warrants further clinical investigation in the treatment of AML patients with high-risk or refractory/relapse diseases, particularly those who are ineligible for intensive chemotherapy.

## Supplementary information

Supplemental Figures 1

Supplemental Figures 2

Supplemental Figures 3

Supplemental Figures 4

Supplemental Figures 5

Supplemental Figures 6

Supplemental Figures 7

Supplemental Figures 8

Supplemental Figure Legends

Supplemental Table S1

Supplemental Table S2

Supplemental Table S3
