# Supplementary figures and images for: Preclinical evaluation of a regimen combining chidamide and ABT-199 in acute myeloid leukemia
Source: Cell Death Dis. 2020 Sep 18;11(9):778. doi: 10.1038/s41419-020-02972-2 (PMC7501858; doi:10.1038/s41419-020-02972-2)

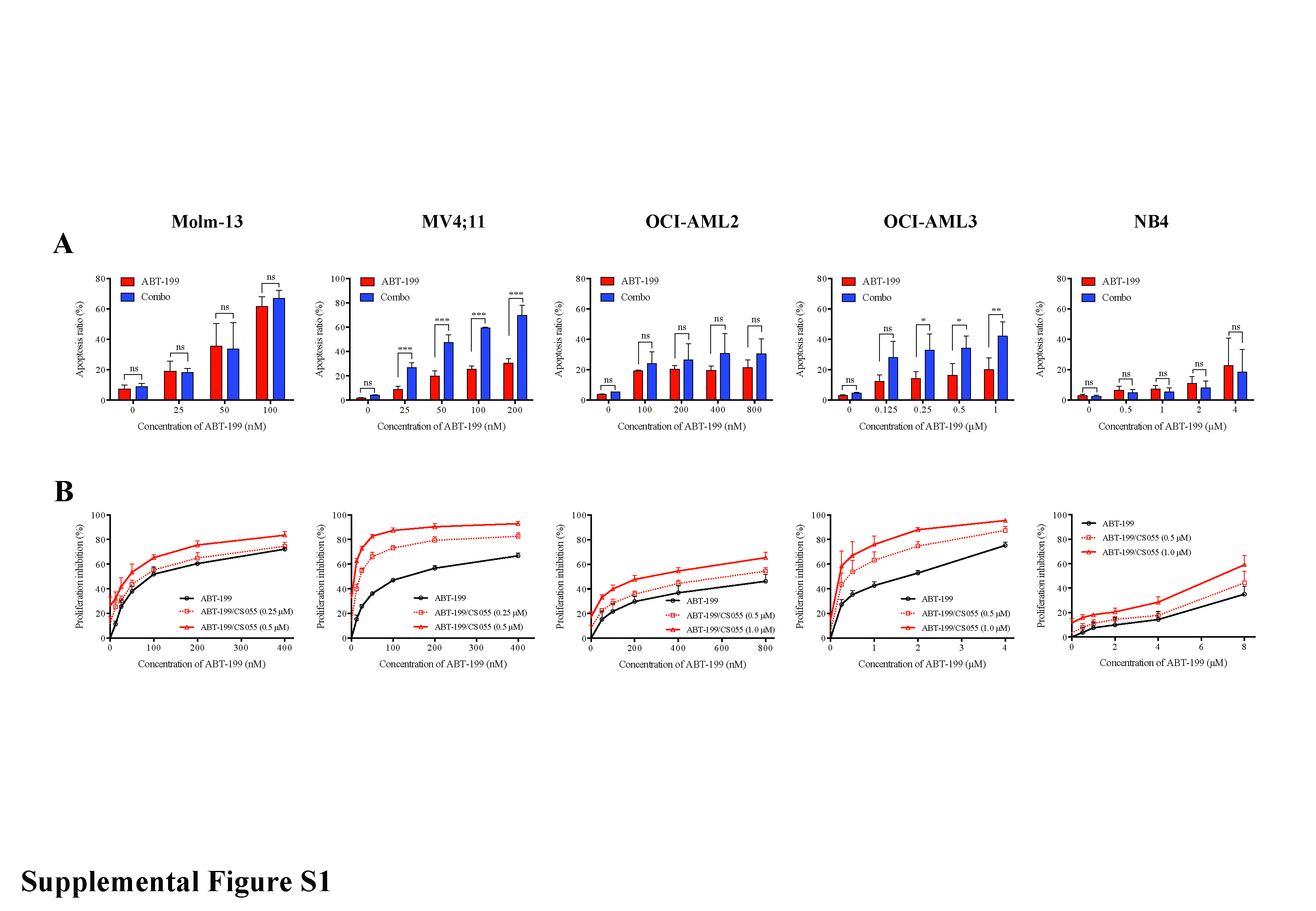

Supplement: Supplementary file 1 — Supplemental Figures 1 [file 41419_2020_2972_MOESM1_ESM.tif]

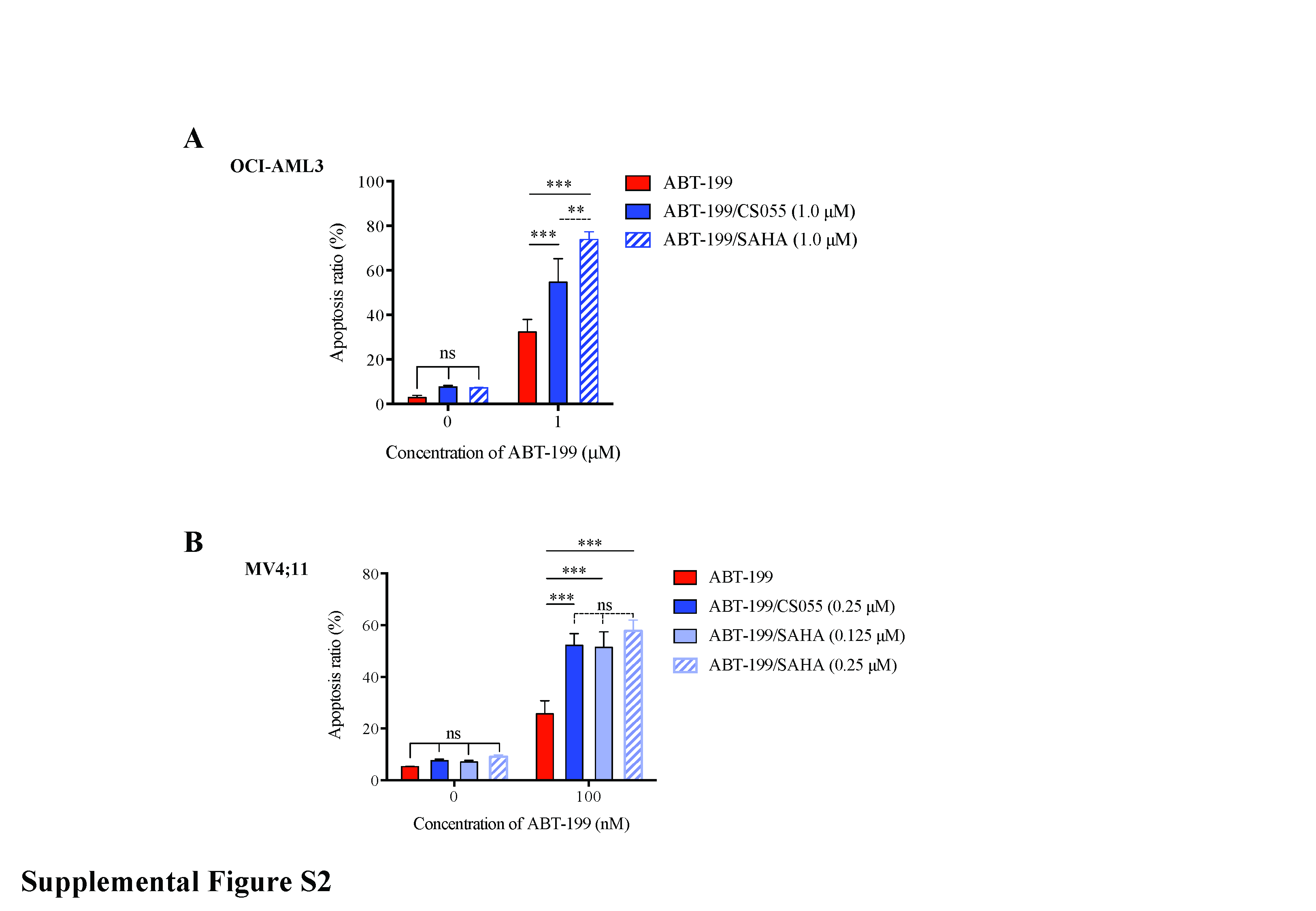

Supplement: Supplementary file 2 — Supplemental Figures 2 [file 41419_2020_2972_MOESM2_ESM.tif]

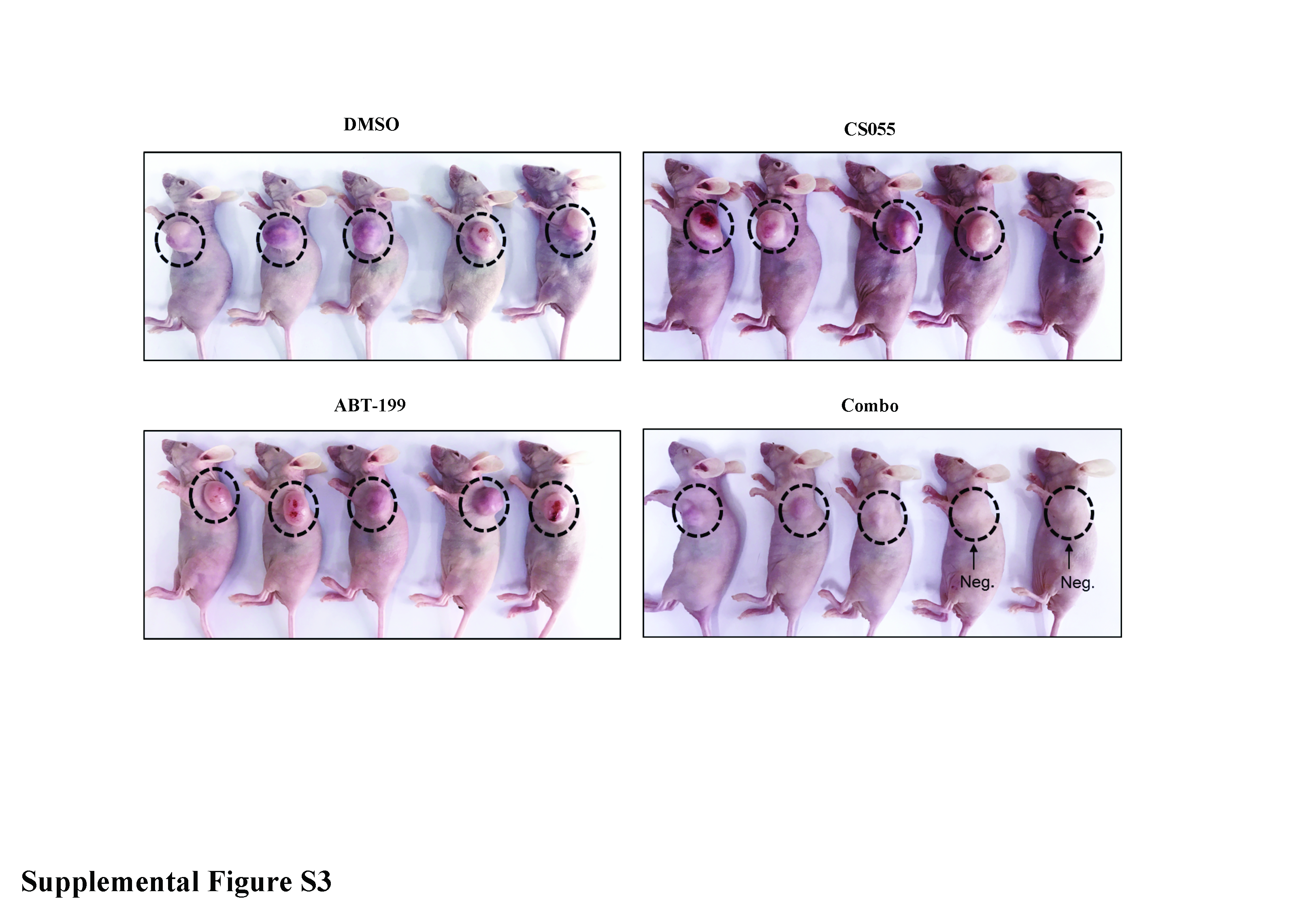

Supplement: Supplementary file 3 — Supplemental Figures 3 [file 41419_2020_2972_MOESM3_ESM.tif]

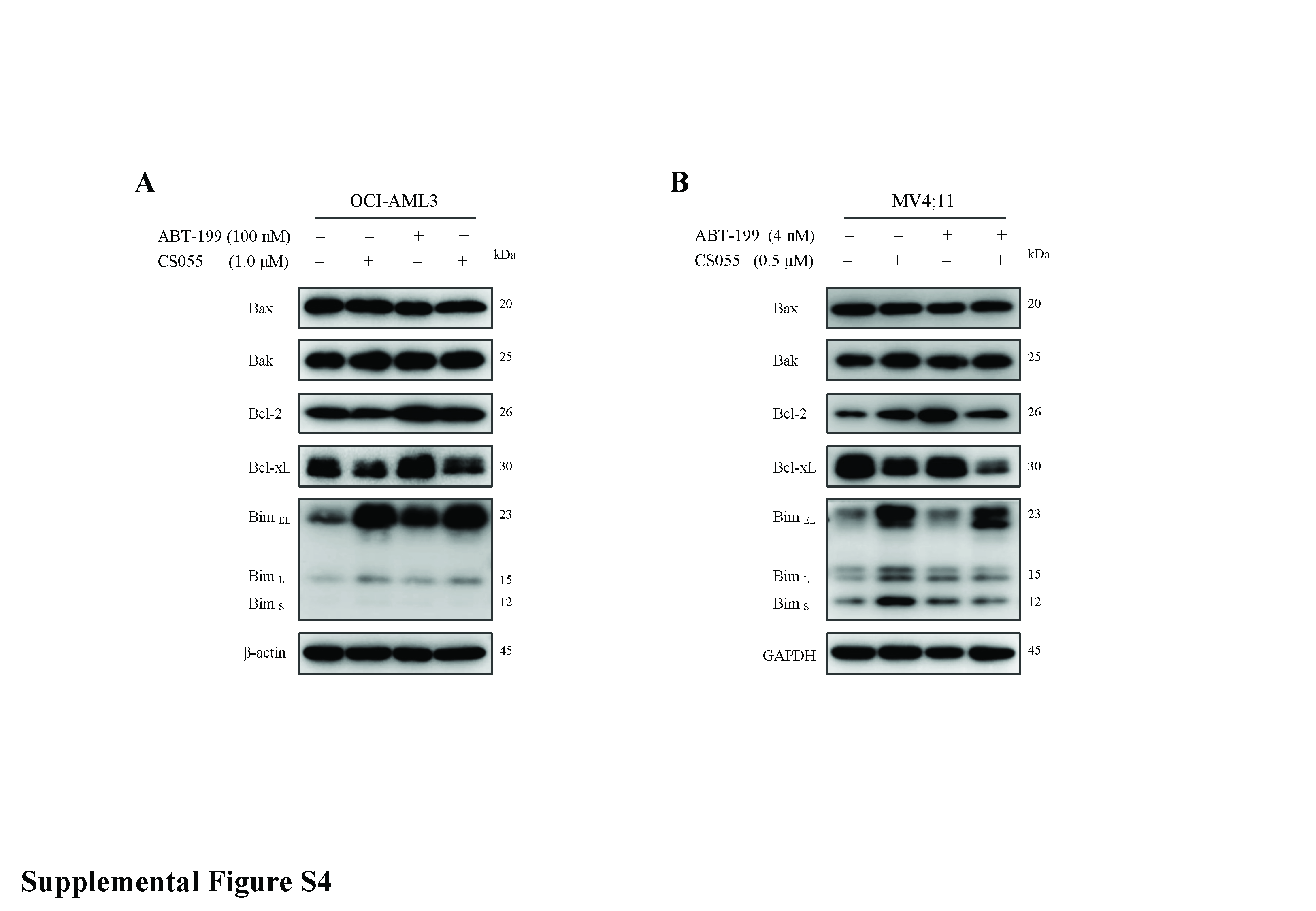

Supplement: Supplementary file 4 — Supplemental Figures 4 [file 41419_2020_2972_MOESM4_ESM.tif]

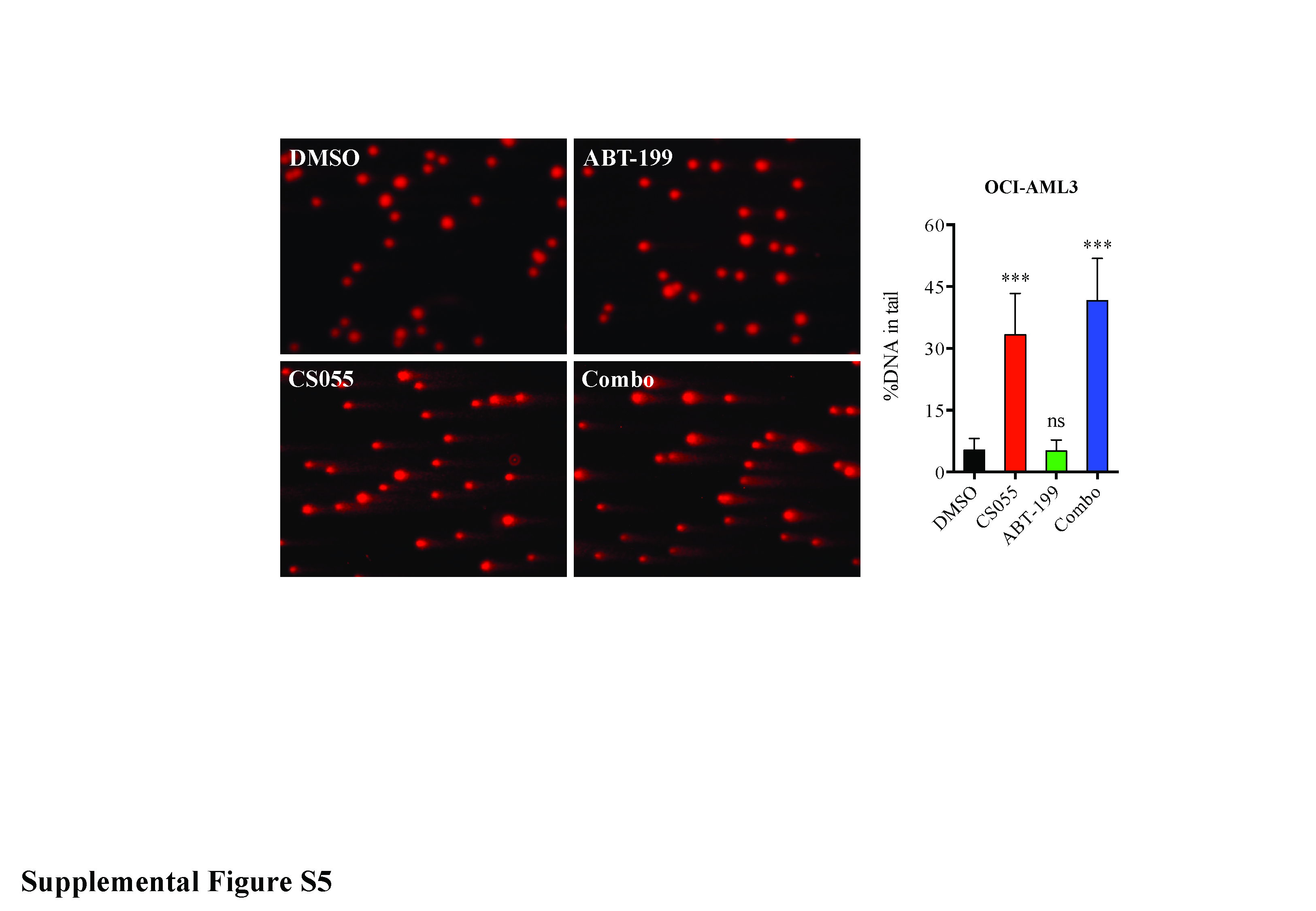

Supplement: Supplementary file 5 — Supplemental Figures 5 [file 41419_2020_2972_MOESM5_ESM.tif]

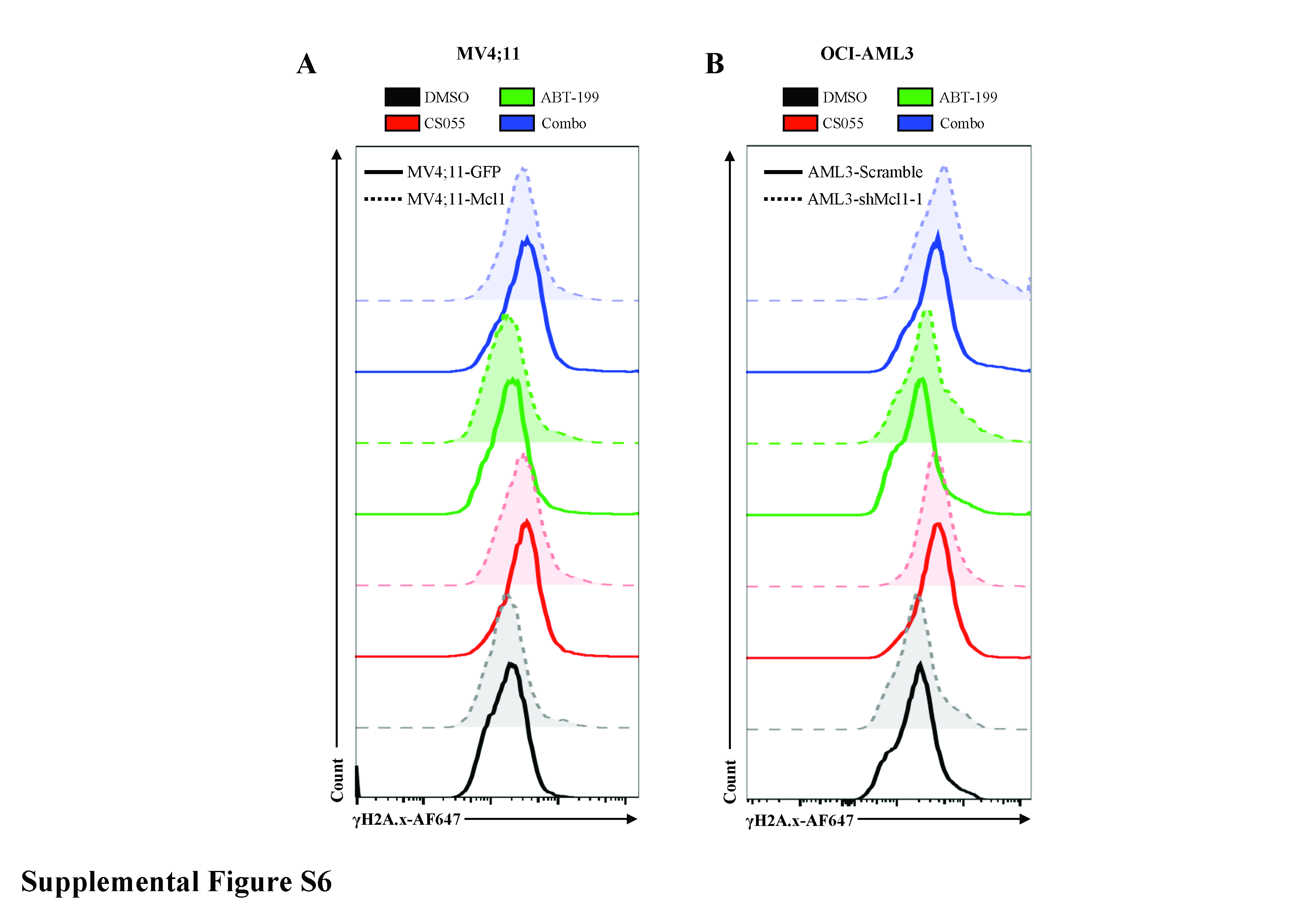

Supplement: Supplementary file 6 — Supplemental Figures 6 [file 41419_2020_2972_MOESM6_ESM.tif]

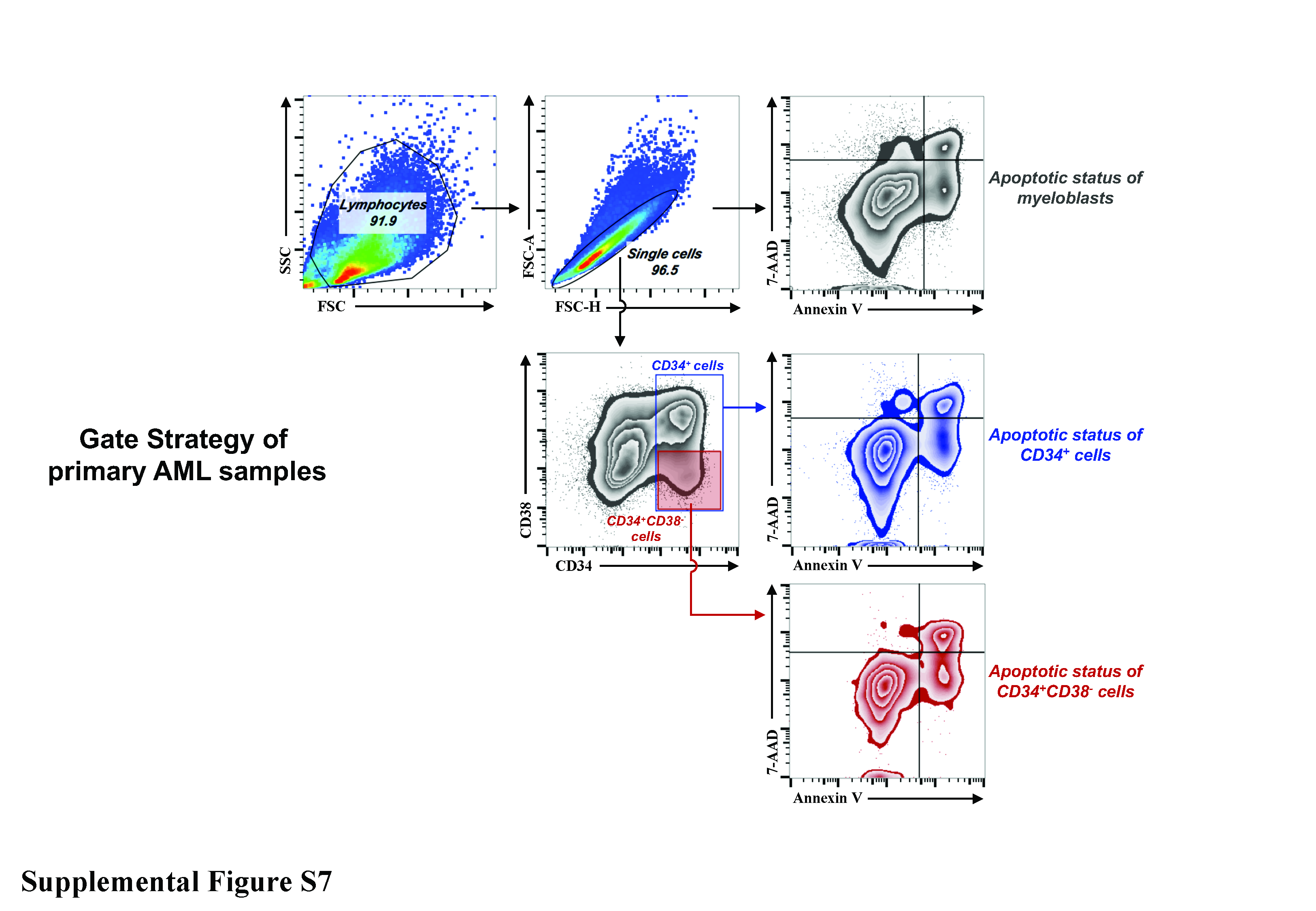

Supplement: Supplementary file 7 — Supplemental Figures 7 [file 41419_2020_2972_MOESM7_ESM.tif]

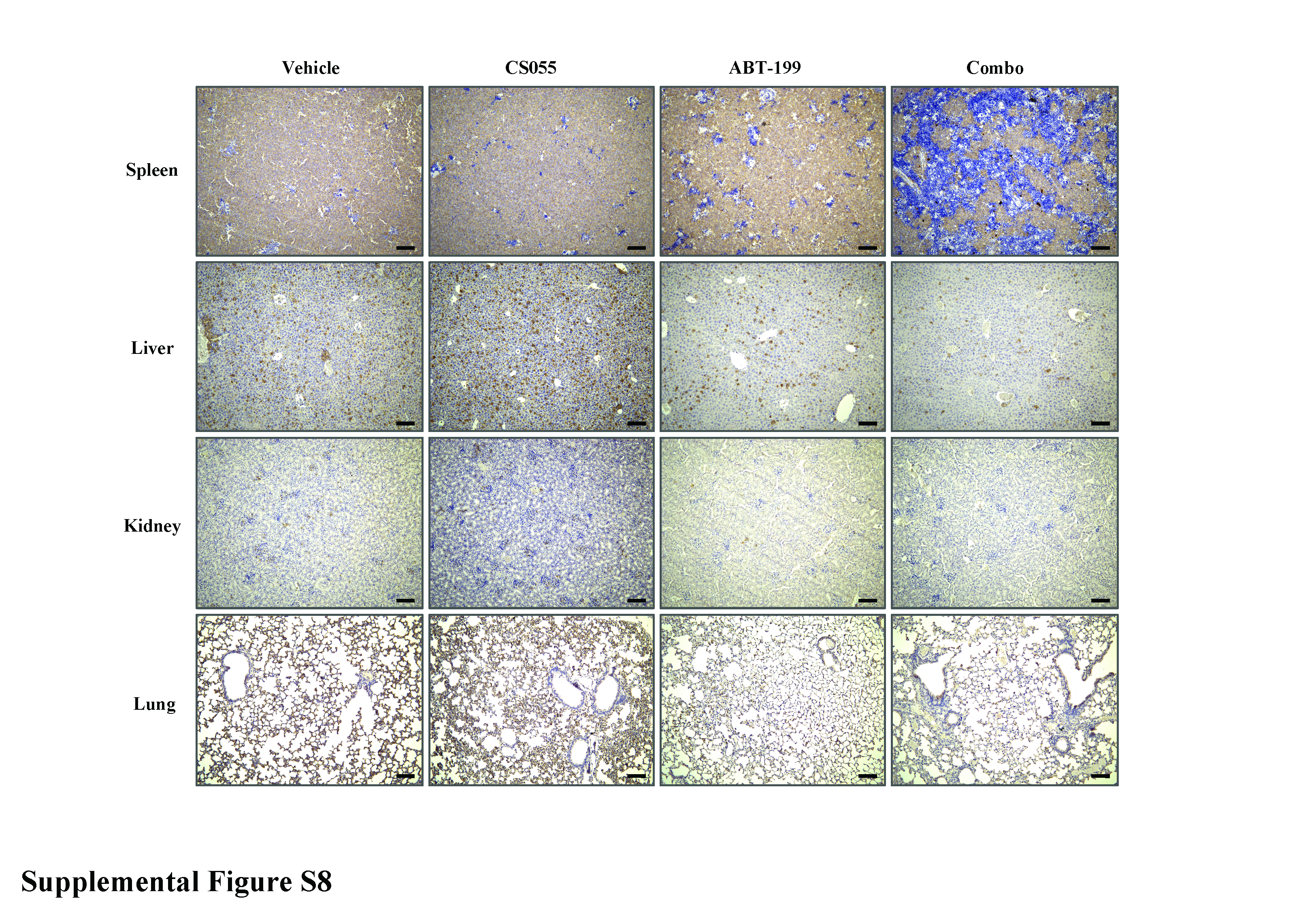

Supplement: Supplementary file 8 — Supplemental Figures 8 [file 41419_2020_2972_MOESM8_ESM.tif]
