# Supplementary material for: Preclinical evaluation of a regimen combining chidamide and ABT-199 in acute myeloid leukemia
Source: Cell Death Dis. 2020 Sep 18;11(9):778. doi: 10.1038/s41419-020-02972-2 (PMC7501858; doi:10.1038/s41419-020-02972-2)
Supplement: Supplementary file 9 — Supplemental Figure Legends [file 41419_2020_2972_MOESM9_ESM.docx]

**Supplemental Figure Legends**

**Figure S1. Analyses of apoptosis and cell viability after 24-hr exposure of AML cells to ABT-199 +/- CS055.** AML cells were exposed to the indicated concentrations of ABT-199 ± CS055 (0.5 μM for Molm-13 and MV4;11, 1.0 μM for OCI-AML2, OCI-AML3, and NB4) for 24 hrs, after which the percentage of apoptotic cells was determined by flow cytometry using Annexin V/PI double staining **(A)**. **(B)** Alternatively, the inhibition rate of cell viability was determined using the CCK-8 kit. Values indicate mean ± SD for at least three independent experiments performed in triplicate (**P*<0.05, ***P*<0.01, ****P*<0.001, ns = not significant).

**Figure S2. SAHA showed a superior sensitization effect only in ABT-199-resistant cell line OCI-AML3, not in ABT-199-sensitive MV4;11. (A)** OCI-AML3 and **(B)** MV4;11 cells were exposed to the indicated concentrations of ABT-199 ± CS055 (1.0 µM for OCI-AML3, and 0.25 µM for MV4;11) or vorinostat (SAHA, 1.0 µM for OCI-AML3, and 0.125 µM, 0.25 µM for MV4;11) for 48 hrs, after which the percentage of apoptotic cells were determined. Sublethal and equivalent dose of CS055 and SAHA was used to yield comparable single agent activity. Values indicate mean ± SD for at least three independent experiments performed in triplicate (***P*<0.01, ****P*<0.001, ns = not significant).

**Figure S3. Representative images for** **the tumor-forming experiment in nude mice.** MV4;11 cells were pre-incubated with ABT-199 ± CS055 for 12 hrs, followed by subcutaneously injection on the left flank of nude mice as described in **Figure 2B-D**. The images were captured after two weeks post cell inoculation (circles indicate tumor-forming area). Two of five mice in the combination group had no visible tumor (Neg = negative).

**Figure S4. CS055 induces the dysregulation of the anti- and pro-apoptotic proteins.** Western blot analysis was performed to monitor expression of the anti- and pro-apoptotic proteins in OCI-AML3 **(A)** and MV4;11 **(B)** cells treated with indicated concentration of ABT-199 ± CS055. Blots were probed for β-actin or GAPDH as loading controls.

**Figure S5. Exposure to CS055 in the presence or absence of ABT-199 induces DNA damage by comet assay.** OCI-AML3 cells were exposed to 100 nM ABT-199 ± 1.0 μM CS055 for 18 hrs, after which the comet assay was performed to examine DNA damage (representative images shown on left). The results were then quantified to determine the percentage of DNA in the comet tail (at least 100 comets; ****P*<0.001, ns = not significant for ABT-199 vs DMSO).

**Figure S6. Mcl-1 does not affect DNA damage induced by CS055 with or without ABT-199. (A)** Mcl-1 was ectopically in MV4;11 cells, after which cells were exposed to 5 nM ABT-199 ± 0.5 μM CS055 for 18 hrs. **(B)** OCI-AML3 cells with shRNA knockdown of Mcl-1 as described in **Figure 3D** (right) were treated with 100 nM ABT-199 ± 1.0 μM CS055 for 18 hrs. After drug treatment, flow cytometry was performed to monitor γH2A.X expression.

**Figure S7. The gating strategy for the analysis of apoptosis in CD34^+^/CD38^-^ leukemic stem/progenitor cells.** The results of this experiment are shown in **Figure 6C-D**.

**Figure S8. Representative images for immunohistochemical analysis of hCD45^+^ cells in multiple organs in the PDX model.** This animal study in a PDX mouse model was described in detailed in **Figure 7**. After drug treatment, mice were sacrificed and immunohistochemical staining for human CD45 were performed to monitor infiltration of leukemic cells in spleen, liver, kidney, and lung (scale bar = 100 µm).
