# Supplementary material for: Preclinical evaluation of a regimen combining chidamide and ABT-199 in acute myeloid leukemia
Source: Cell Death Dis. 2020 Sep 18;11(9):778. doi: 10.1038/s41419-020-02972-2 (PMC7501858; doi:10.1038/s41419-020-02972-2)
Supplement: Supplementary file 10 — Supplemental Table S1 [file 41419_2020_2972_MOESM10_ESM.docx]

**Table S1. IC_50_ values of ABT-199 alone or in combination with CS055 in AML cell lines**

| AML cell lines | Mutations | IC_50_ (nM) at 24 hr | | | | | IC_50_ (nM) at 48 hr | | | | |
| --- | --- | --- | --- | --- | --- | --- | --- | --- | --- | --- | --- |
|  |  | ABT-199 | ABT-199  + CS055^a^ | Fold | ABT-199  + CS055^b^ | Fold | ABT-199 | ABT-199  + CS055^a^ | Fold | ABT-199  + CS055^b^ | Fold |
| Molm-13 | FLT3 mut; t(9;11) | 109.27 | 116.67 | 0.937 | 101.32 | 1.078 | 32.55 | 14.05 | 2.317 | 6.57 | 4.954 |
| MV4;11 | FLT3 mut | 130.36 | 34.29 | 3.802 | 13.68 | 9.529 | 46.08 | 3.49 | 13.203 | 0.25 | 184.32 |
| OCI-AML2 | DNMT3A mut | 1019.34 | 801.09 | 1.272 | 496.71 | 2.052 | 1014.84 | 427.16 | 2.376 | 180.50 | 5.622 |
| OCI-AML3 | NRAS mut; DNMT3A mut | 1206.27 | 483.45 | 2.495 | 300.16 | 4.019 | 1202.14 | 258.22 | 4.655 | 123.20 | 9.758 |
| NB4 | t(15;17) | >10,000 | >10,000 | – | >10,000 | – | 4285.85 | 2023.88 | 2.118 | 1147.95 | 3.733 |

1. IC_50_: half maximal inhibitory concentration, determined by the CCK-8 assay.

2. ^a^ and ^b^ indicate low and the high doses of CS055 described in **Figure 1**, respectively.

3. IC_50_ values for each combination group were calculated after the inhibition rate of CS055 was normalized following the formula as 100 × (% inhibition rate of combo treated cells – % inhibition rate of CS055-treated cells)/ (100 – % inhibition rate of CS055-treated cells).
