# Supplementary material for: Preclinical evaluation of a regimen combining chidamide and ABT-199 in acute myeloid leukemia
Source: Cell Death Dis. 2020 Sep 18;11(9):778. doi: 10.1038/s41419-020-02972-2 (PMC7501858; doi:10.1038/s41419-020-02972-2)
Supplement: Supplementary file 11 — Supplemental Table S2 [file 41419_2020_2972_MOESM11_ESM.docx]

**Table S2. Clinical characteristics of AML patients enrolled into this study (n=36)**

| **No.** | **Disease**  **Status** | **Age/**  **Gender** | **FAB**  **subtype** | **WBC**  **(×10^9^/L)** | **PB blasts (%)** | **BM blasts (%)** | **LDH (U/L)** | **Karyotype** | **Mutation** |
| --- | --- | --- | --- | --- | --- | --- | --- | --- | --- |
| 1 | *De novo* | 45/M | M2 | 31.47 | 58% | 59.5% | 1036 | 45, X, -Y t(8;21)(q22;q22)[4] | AML1/ETO, WT1 |
| 2 | *De novo* | 62/M | M2 | 107.34 | 88.0% | 88.5% | 777 | U | CBFβ-MYH11, C-kit, WT1 |
| 3 | Refractory | 38/M | M2a | 26.94 | 61.0% | necrosis | 5350 | 46, XY[20] | NRAS, PHF6, TET2, TP53 |
| 4 | Relapse | 30/M | M2a | 135.50 | 43.0% | 56.0% | 396 | U | U |
| 5 | *De novo* | 39/M | M1 | 101.33 | 88.0% | 94.0% | 387 | U | IDH1, WT1 |
| 6 | Relapse | 83/M | CMML-M5 | 85.52 | 52.0% | 59.0% | 1453 | 46, XY[20] | WT1 |
| 7 | Relapse | 65/M | M2 | 7.09 | 14.0% | 24.0% | 333 | 46, XY[20] | DNMT3A, NPM1, FLT3-ITD |
| 8 | *De novo* | 75/M | M4 | 44.08 | 17.0% | 31.0% | 609 | 46, XY[20] | TET2, KMT2A, NPM1, FLT3-ITD |
| 9 | *De novo* | 34/F | M5b | 45.26 | 53.0% | 85.5% | 206 | 46, XX[20] | NPM1, FLT3-ITD, WT1 |
| 10 | *De novo* | 39/M | M2 | 9.23 | 74.0% | 53.5% | 117 | 46, XY[20] | CEBPA |
| 11 | *De novo* | 23/F | M5b | 1.41 | 13.0% | 43.0% | 395 | 45, XX, del(14)(q31), -21, add(22)(p13)[20] | Normal |
| 12 | *De novo* | 39/F | M5b | 6.30 | 23.0% | 67.0% | 489 | 46, XX, i(17)(q10)[6]/47, idem, +22[14] | CBFβ-MYH11, WT1 |
| 13 | Relapse | 25/F | M1 | 4.83 | 65.0% | 71.5% | 160 | 46, XX, t(2;9)(p13;q34)[15]/46, XX[5] | FLT3-ITD, WT1 |
| 14 | *De novo* | 43/F | M4EO | 1.87 | 1.0% | 24.0% | 175 | 46, XX[20] | WT1 |
| 15 | Relapse | 39/F | M5b | 127.57 | 81.0% | 87.5% | 406 | U | MLL-AF9, WT1 |
| 16 | *De novo* | 66/F | M5b | 55.42 | 51.0% | 41.0% | 806 | 46, XX[10] | WT1 |
| 17 | *De novo* | 58/F | M2 | 3.00 | 6.0% | 45.5% | 255 | 46, XX[20] | DNMT3A, WT1 |
| 18 | *De novo* | 56/M | M2b | 71.08 | 32.0% | 49.5% | 864 | 45, X, -Y, t(8;21)(q22;q22)[20] | AML1/ETO, CEBPA |
| 19 | *De novo* | 62/F | M2 | 8.23 | 20.0% | 29.5% | 341 | 46, XX[20] | DNMT3A, NPM1, IDH1, ASXL1, JAK2, PTEN |
| 20 | Relapse | 34/F | M5b | 216.43 | - | - | 1111 | 46, XX[20] | NPM1, FLT3-ITD, WT1 |
| 21 | *De novo* | 51/M | M2 | 62.12 | 69.0% | 34.0% | 933 | 46, XY[20] | CEBPA |
| 22 | *De novo* | 45/M | M2 | 211.80 | 44.0% | 84.0% | 1236 | 46, XY[20] | NPM1, FLT3-ITD |
| 23 | *De novo* | 27/F | M4 | 40.49 | 79.0% | 62.5% | 322 | U | U |
| 24 | *De novo* | 21/F | M4EO | 225.66 | 76.0% | 86.0% | 1466 | 46, XX, inv(16)(p13;q22)[20] | CBFβ-MYH11 |
| 25 | Refractory | 71/M | MDS-AML | 4.91 | 20.0% | 22.5% | 197 | U | JAK2 V617F |
| 26 | Refractory | 74/M | MDS-AML | 3.61 | 8% | 31.5% | 269 | 42, XY, +del(1)(p31p36.1), +add(4)(q21), del(5)(q22q31), -6, -7, -7, add(9)(p13), der(10)t(10;15)(p13;q21), -13, -15, add(16)(q24), -22, -22, +mar[17]/46,XY[3] | TET2 |
| 27 | *De novo* | 10/F | M5b | 471.38 | 89.0% | 92.5% | 1655 | U | U |
| 28 | *De novo* | 57/M | M2 | 16.79 | 20.0% | 20.5% | 233 | 45, X, -Y, t(8;22;21)(q22;q13;q22)[20] | AML1/ETO, TET2, NRAS, WT1 |
| 29 | *De novo* | 11/F | M5b | 12.99 | 79.0% | 81.5 | 1718 | U | U |
| 30 | *De novo* | 29/F | M2a | 70.02 | 76.0% | 80.0% | 247 | 46, XX[20] | NPM1, NRAS |
| 31 | *De novo* | 66/M | M2 | 1.38 | 40.0% | 58.5% | 383 | 46, XY[20] | IDH2, ASXL1, WT1 |
| 32 | *De novo* | 45/M | M2 | 29.93 | 88.0% | 72.5% | 325 | 46, XY[20] | CEBPA |
| 33 | Refractory | 69/F | MDS-AML | 1.61 | 1.0% | 22.5% | 320 | 47, XX, +8[20] | TET2, SRSF2 |
| 34 | *De novo* | 42/F | M2 | 89.34 | 46.0% | 86.0% | 528 | 46, XX[20] | NPM1, IDH2, WT1 |
| 35 | *De novo* | 45/F | M2a | 43.72 | 93.0% | 73.0% | 230 | 46, XX[16] | CEBPA |
| 36 | Relapse | 27/M | M2 | 19.17 | 82.0% | 93.0% | 2719 | 47, XY,+8[12] | Normal |
