# Supplementary material for: Preclinical evaluation of a regimen combining chidamide and ABT-199 in acute myeloid leukemia
Source: Cell Death Dis. 2020 Sep 18;11(9):778. doi: 10.1038/s41419-020-02972-2 (PMC7501858; doi:10.1038/s41419-020-02972-2)
Supplement: Supplementary file 12 — Supplemental Table S3 [file 41419_2020_2972_MOESM12_ESM.docx]

**Table S3. The relationship between clinical characteristics of AML patients and *ex vivo* sensitivity of primary samples to ABT-199 ± CS055**

| **Characteristics** | **Subgroups** | **Specific apoptosis^†^ (%)** | | | | ***P* value** |
| --- | --- | --- | --- | --- | --- | --- |
|  |  | DMSO  (normalized) | CS055  (1.0 μM) | ABT-199  (25 nM) | ABT-199  + CS055 |  |
| AML1/ETO | Negative (n=33) | 0 | 12.04±10.54 | 23.89±18.71 | 43.43±22.42 | *0.2324* |
|  | Positive (n=3) | 0 | 3.21±5.97 | 25.29±17.21 | 28.67±13.76 |  |
| CBFβ-MYH11 | Negative (n=33) | 0 | 11.35±10.85 | 24.43±19.14 | 42.04±23.00 | *0.8465* |
|  | Positive (n=3) | 0 | 10.83±6.00 | 19.34±2.78 | 44.02±7.71 |  |
| NRAS | Wt (n=33) | 0 | 11.60±10.43 | 24.28±18.46 | 41.65±22.97 | *0.9936* |
|  | Mut (n=3) | 0 | 8.03±12.67 | 21.05±20.91 | 48.29±5.70 |  |
| TET2 | Wt (n=31) | 0 | 11.92±10.67 | 25.30±18.49 | 43.65±22.76 | *0.1040* |
|  | Mut (n=5) | 0 | 7.49±9.24 | 16.02±17.19 | 33.19±16.20 |  |
| IDH1/2 | Wt (n=32) | 0 | 11.41±10.28 | 24.13±18.53 | 42.27±21.10 | *0.8689* |
|  | Mut (n=4) | 0 | 10.47±13.68 | 23.00±19.65 | 41.63±32.96 |  |
| DNMT3A | Wt (n=33) | 0 | 11.54±10.86 | 24.88±18.89 | 42.98±22.91 | *0.2246* |
|  | Mut (n=3) | 0 | 8.75±4.54 | 14.4±6.43 | 33.60±3.89 |  |
| FLT3-ITD | Wt (n=30) | 0 | 10.60±10.74 | 25.35±18.61 | 42.53±22.10 | *0.6751* |
|  | Mut (n=6) | 0 | 14.85±8.97 | 17.30±16.97 | 40.53±24.02 |  |
| NPM1 | Wt (n=28) | 0 | 10.22±10.35 | 24.80±18.41 | 40.60±21.20 | *0.4904* |
|  | Mut (n=8) | 0 | 15.11±10.68 | 21.24±19.16 | 47.80±25.67 |  |
| CEBPA | Wt (n=31) | 0 | 11.19±10.24 | 22.61±17.49 | 41.51±20.50 | *0.2876* |
|  | Mut (n=5) | 0 | 11.99±13.15 | 32.67±23.40 | 46.49±32.99 |  |
| Hyperleukocytosis | WBC<100 ×10^9^/L (n=28) | 0 | 10.65±10.58 | 22.39±18.13 | 38.66±22.38 | ***0.0323**** |
|  | WBC>100 ×10^9^/L (n=8) | 0 | 13.60±10.43 | 29.68±19.25 | 54.58±16.75 |  |
| AML FAB subtype | M1 (n=2) | 0 | 7.51±3.03 | 12.54±1.65 | 32.61±0.36 | *0.5076* |
|  | M2 (n=18) | 0 | 12.41±12.50 | 28.22±20.55 | 45.83±23.83 |  |
|  | M4 (n=4) | 0 | 15.15±6.99 | 24.08±19.58 | 50.23±18.85 |  |
|  | M5 (n=8) | 0 | 11.01±10.17 | 24.10±16.53 | 44.41±21.48 |  |
| Disease type | Primary AML(n=32) | 0 | 12.09±10.82 | 25.69±18.61 | 45.20±21.54 | ***0.002***** |
|  | Secondary AML (n=4) | 0 | 4.99±3.23 | 10.53±9.21 | 18.21±5.71 |  |
| Risk stratification^††^ | Favorable risk (n=10) | 0 | 12.79±11.30 | 27.42±20.11 | 48.01±26.91 | *0.3404* |
|  | Intermediate risk (n=16) | 0 | 9.48±10.95 | 26.44±17.89 | 40.89±20.22 |  |
|  | High risk (n=10) | 0 | 12.74±9.41 | 16.70±17.06 | 38.49±20.85 |  |

^†^Specific apoptosis rate was calculated by the formula as 100 × (% apoptosis of drug treated cells – % apoptosis of DMSO-treated control cells)/ (100 – % apoptosis of DMSO-treated control cells). ^††^Risk stratification was evaluated according to the AML NCCN Guidelines (Version 1.2016).
